# Prognostic Value of Multiple Manual Segmentation Methods for Diffuse Large B-Cell Lymphoma with ^18^F-FDG PET/CT

**DOI:** 10.3390/curroncol32060356

**Published:** 2025-06-16

**Authors:** Andrej Doma, Andrej Studen, Barbara Jezeršek Novaković

**Affiliations:** 1 Department of Nuclear Medicine, Institute of Oncology Ljubljana, 1000 Ljubljana, Slovenia; 2 Faculty of Medicine, University of Ljubljana, 1000 Ljubljana, Slovenia; 3 Experimental Particle Physics Department, Jožef Stefan Institute, 1000 Ljubljana, Slovenia; 4 Faculty of Mathematics and Physics, University of Ljubljana, 1000 Ljubljana, Slovenia; 5 Division of Medical Oncology, Institute of Oncology Ljubljana, 1000 Ljubljana, Slovenia

**Keywords:** diffuse large B-cell lymphoma, ^18^F-FDG, PET/CT, MTV, TLG, overall survival, segmentation, threshold

## Abstract

Quantitative ^18^F-FDG PET/CT-derived metabolic metrics are strongly associated with patient outcomes in diffuse large B-cell lymphoma (DLBCL), but the lack of consensus on optimal segmentation thresholds limits standardization. This study evaluated the prognostic value of various metabolic tumor volume (MTV) segmentation approaches in 140 stage II–IV DLBCL patients treated with standard immunochemotherapy. MTV was derived using fixed SUV (≥2.5, ≥4.0), relative (>41% SUVmax), and adaptive (liver-to-background) thresholds. Baseline MTV metrics significantly correlated with 3-year overall survival (OS3) in univariate analysis in overall cohort, with MTV41 showing the strongest association (HR: 1.27; *p* = 0.003). MTV25 and MTV41 remained significant in the stage 4 patient subgroup. However, in multivariate analysis, no MTV metric independently predicted OS3 when adjusted for the International Prognostic Index (IPI), which remained the dominant predictor (HR: 1.95; *p* < 0.0001). ROC analysis confirmed superior AUC for IPI (0.76) over PET-based metrics (0.64–0.69). Predictive models integrating IPI with PET metrics were robust but failed to improve prognostic accuracy beyond IPI alone. Although PET-derived MTV metrics provide prognostic value in univariate analysis, threshold selection has minimal impact, and their added value is limited when combined with IPI, reinforcing its role as the most reliable survival predictor in DLBCL.

## 1. Introduction

Diffuse large B-cell lymphoma (DLBCL) is the most common subtype of aggressive non-Hodgkin lymphoma in adults in the Western world [1,2], with annual incidence rates of 5.6 per 100,000 person–years in the United States [3] and 6.33 per 100,000 in Slovenia [4]. Initial treatment with rituximab, cyclophosphamide, doxorubicin, vincristine, and prednisone (R-CHOP) leads to complete remission (CR) in over 75% of patients with advanced-stage diseases, and among those who remain event-free for 24 months posttreatment, mortality rates eventually align with those of the general population [5,6]. However, 10–15% of patients experience primary treatment failure, and 20–25% relapse, with salvage therapies often yielding limited success [7,8]. Therefore, accurate prognostic markers are essential for identifying high-risk patients who may benefit from more intensive or novel therapeutic strategies applied in first treatment [9].

^18^F-Fluorodeoxyglucose (FDG) Positron Emission Tomography-Computed Tomography (PET/CT) is routinely used to assess metabolic activity across all lymphoma lesions [10]. High baseline metabolic tumor burden, as measured by metabolic tumor volume (MTV) and total lesion glycolysis (TLG) on PET/CT, is associated with poor prognosis in DLBCL [11,12,13,14] and has been reported to outperform conventional international prognostic scores [15,16,17,18]. However, there is no standardized method for calculating MTV, with several different thresholding techniques currently in use [19]. While accurate manual segmentation of FDG PET/CT uptake is time-consuming and subject to interobserver variability [9], several user-friendly and efficient automated segmentation tools often struggle to differentiate between tumor and physiological uptake, making full automation unreliable [20]. The most common approaches for manual delineation to assess MTV on PET/CT include the following: fixed Standardized Uptake Value (SUV) thresholds (e.g., SUV ≥ 2.5 g/mL or SUV ≥ 4.0 g/mL), a relative SUV threshold (e.g., 41% of SUVmax) [9], and adaptive threshold methods like the ratio between lesion SUVmax and liver SUVmean [21]. MTVs based on fixed thresholds of SUV ≥ 4.0 g/mL (MTV4) and SUV ≥ 2.5 g/mL (MTV25) are relatively straightforward to derive from, as these thresholds provide clear and consistent limits for lesion delineation. In contrast, deriving a MTV using a relative threshold of SUV > 41% SUVmax (MTV41) or a liver activity-adjusted threshold, such as 1.5 × liver SUVmean + 2 standard deviations (MTVSD15), is more demanding, requiring first to determine the SUVmax, and then to calculate the specific threshold for delineation of the tumor. To address this complexity, an alternative approach could be to estimate regression-derived MTV41′ variable using linear regression models based on fixed thresholding methods, such as MTV25, MTV4, and MTVSD15 as reference variables.

The aim of this study was to evaluate the prognostic value of baseline PET/CT-derived MTV in patients with stage II–IV DLBCL using various commonly applied thresholding methods. Specifically, we sought to determine (1) whether selection of abnormal uptake threshold on baseline PET/CT bears any impact on success of survival prediction; (2) how the prognostic performance of MTV metrics compares to conventional prognostic tools such as the International Prognostic Index (IPI) and its individual constituents, including stage, lactate dehydrogenase (LDH), along with MIB-1 immunohistochemical proliferation index (MIB-1); (3) how IPI influences prognostic outcomes when combined with PET/CT metrics; (4) whether regression-derived variable MTV41′ from fixed threshold-derived MTVs provide a simpler, reliable, and more practical alternative to the original MTV41, therefore omitting the need for additional manual determination of SUVmax and associated specific thresholds.

## 2. Materials and Methods

The study included 140 consecutive patients with histologically confirmed stage II–IV DLBCL with pretreatment FDG PET/CT performed, followed by treatment at the Institute of Oncology Ljubljana from January 2016 to December 2020. All patients were part of a previously published study on the prognostic value of PET-derived tumor burden [22].

Written informed consent was obtained from all participants for undergoing PET/CT imaging, bone marrow biopsy (BMB), and for the use of their medical data for research purposes. The study received ethical approval from both the Institutional Ethics Committee of the Institute of Oncology Ljubljana (approval number: ERIDEK-0104/2019) and the National Medical Ethics Committee of the Republic of Slovenia (approval number: 0120-104/2021/3).

Participants were excluded if they were younger than 18 or older than 80 years, had stage I disease, central nervous system (CNS) involvement, or a history of concurrent or prior malignancies—including low-grade lymphoma, which was ruled out through a mandatory pretreatment BMB. Treatment consisted of 6 or 8 cycles of R-CHOP or an equivalent regimen in accordance with local clinical guidelines, with radiotherapy administered to residual disease when indicated.

Progression-free survival at two years (PFS2) was calculated from the initiation of treatment to the date of disease progression, relapse, death from any cause, or last follow-up, depending on which occurred first. Overall survival (OS) was calculated from the initiation of treatment to the date of censoring, last follow-up, or death from any cause. No distinction was made between deaths directly attributable to DLBCL and those resulting from unrelated causes. Three-year overall survival (OS3) rates were determined.

PET/CT imaging was conducted using a Siemens Biograph mCT40 scanner (Siemens Healthineers, Erlangen, Germany), following the protocols outlined by the European Association of Nuclear Medicine (EANM) [23]. Patients fasted for at least 6 h, with blood glucose < 11 mmol/L (IV insulin if needed), received 3.7 MBq/kg ^18^F-FDG IV one hour prior to scanning, and underwent head-to-mid-thigh PET/CT with arms raised. Acquisition parameters included an automatic adjustment of tube current and voltage based on a reference setting of 100 kV and 80 mAs, a beam width of 16 × 1.2 mm, a pitch of 1.2, and a PET acquisition duration of 2 min per bed position.

Pretreatment ^18^F-FDG PET/CT scans were interpreted and segmented by a board-certified nuclear medicine physician with 15 years of experience in oncological PET/CT imaging. Quantitative measurements, including MTV, total lesion glycolysis (TLG), and SUVmax, were extracted using the 3D Slicer image processing platform (version 5.0.2, http://www.slicer.org) [24], incorporating the pyRadiomics feature extraction tool. These metrics were derived from shape and first-order features, specifically volume, energy, and maximum value, respectively [25,26].

All DLBCL lesions were manually segmented using 3D isocontour-based volumes of interest (VOIs) defined by a fixed SUV threshold of ≥2.5 g/mL, with no minimum volume constraint. Slices were reviewed in multiple planes to ensure the segmentation conformed to known patterns of lymphomatous involvement and avoided nonmalignant tissue. Bone marrow infiltration (BMI) was defined as focal or multifocal FDG uptake on PET/CT exceeding liver activity, not attributable to benign causes by CT or clinical assessment. Confirmation of BMI required concordance with iliac crest biopsy or resolution upon follow-up posttreatment PET/CT. Negative BMI included diffuse or absent uptake, benign etiology, or persistent activity detected on evaluation scans. Splenic infiltration was defined by FDG uptake higher than liver SUVmax activity, irrespective of the uptake pattern, and without clinical or historical evidence of systemic inflammation or infection. Necrotic regions within lesions were included in the segmentation if they fell within the defined isocontour boundaries; however, they were included in specific MTVs only if their uptake exceeded the SUV threshold applied for that particular MTV method. Particular care was taken to distinguish malignant lesions from areas of physiological or inflammatory FDG uptake, which could mimic disease involvement. Common sites of physiological activity, such as the brain, myocardium, kidneys, bowel, and urinary tract, were carefully excluded, while physiological uptake in salivary glands, tonsils, thymus, gastrointestinal tract, genitourinary system, and brown adipose tissue was identified based on their characteristic anatomical locations, symmetrical distribution, and lack of corresponding structural abnormalities on CT. Inflammatory uptake, potentially related to infections, recent biopsy sites, or reactive lymph nodes, was evaluated in the clinical context and excluded if morphological features or clinical history suggested a benign etiology. Whenever uncertainty arose, consensus was sought through correlation with clinical data and follow-up imaging when available.

Additionally, spherical VOIs measuring 1 cm and 5 cm in diameter were placed in the aortic arch and the right upper lobe of the liver, respectively, to determine background physiological uptake by calculating the mean SUV (SUVmean) in the blood and liver. Metabolic tumor volume was computed as the cumulative volume of all DLBCL lesions exceeding the threshold, using multiple segmentation approaches: MTV25, MTV4, MTV41, and MTVSD15. The lesion-to-liver ratio (LLR) was defined as the ratio between the lesion SUVmax and the liver SUVmean. Additionally, SUVmax and IPI score were recorded for comparison. TLG was calculated by multiplying the MTV by the SUVmean of all VOIs.

To compare the original MTV41 values with regression-derived equivalents, we extracted regression-derived values for MTV41′, using linear regression equations based on MTV25, MTV4, and MTVSD15; these newly derived variables are referred as MTV41′ (MTV25), MTV41′ (MTV4), and MTV41′ (MTVSD15), respectively. We derived equivalent values denoted by primes by constructing a simple linear regression model. In all cases, MTV41 was taken as the dependent variable, and MTV25, MTVSD15, and MTV4 were taken as independent variables in three independent, single explanatory variable models. The models were fit using data for all participants to yield independent variable-related slopes β_MTV25_, β_MTVSD15_, and β_MTV4,_ respectively, and intercepts α_MTV25_, α_MTVSD15_, and α_MTV4_. The corresponding equivalent values were calculated as MTV41′ (X)= β_X_ X + α_X_, where X can be any of MTV25, MTVSD15, or MTV4.

Statistical analyses were performed using MedCalc (v19.2.6, MedCalc Software, Ostend, Belgium). Pearson’s R assessed variable correlations. Median MTVs from different thresholding methods were compared via linear regression (R^2^) and Bland–Altman plots. Receiver-operating characteristic (ROC) curves were calculated for variables identified to bear statistical significance and the multinomial logistic regression model. Predictive power was compared using area under ROC curve (AUC), pairwise comparisons for statistical significance of the differences between AUCs were conducted using DeLong’s test [27]. Sensitivity and specificity were reported at optimal cutoffs determined by Youden’s index, which also defined thresholds for odds ratios and Kaplan–Meier analysis. Survival curves were compared using the log-rank test. Univariate and multivariate Cox regression were performed to assess PET volumetric parameters and IPI. Statistical significance was set at *p* < 0.05.

## 3. Results

Medical records of 507 patients were retrospectively reviewed. DLBCL was histologically confirmed in 371 patients. Of those, 229 patients were excluded from analysis (stage I disease n = 33; CNS involvement n = 15; palliative care n = 11; no PET/CT performed n = 126; no baseline BMB performed n = 28; age > 80 years n = 16). The PET/CT DICOM images of two additional patients could not be retrieved. Thus, 140 patients were included in the analysis. Patients’ characteristics are presented in Table 1.

The IPI distribution in the overall cohort (IPI 0 to 5, n = 140) was nonuniform (χ^2^ = 17.4, *p* = 0.004), mainly due to very few IPI 0 cases and an excess of IPI 3 and 4 cases. Among the 84 stage 4 patients analyzed separately, IPI classes were limited to 2 to 5 (Supplement S4, Table S4), and the distribution was close to uniform (χ^2^ = 7.6, *p* = 0.055).

The median values of SUVmax, MTV25, MTV4, MTV41, MTVSD15, and LLR in the overall cohort were 26.8, 331.1 mL, 224.9 mL, 64.5 mL, 225.3 mL, and 12.9, respectively (Table 2), compared with 27.9, 652.4 mL, 410.6 mL, 85.5 mL, 398.6 mL, and 13.5 in the stage 4 patient subgroup (Supplement S3, Table S3).

**Figure 1:**
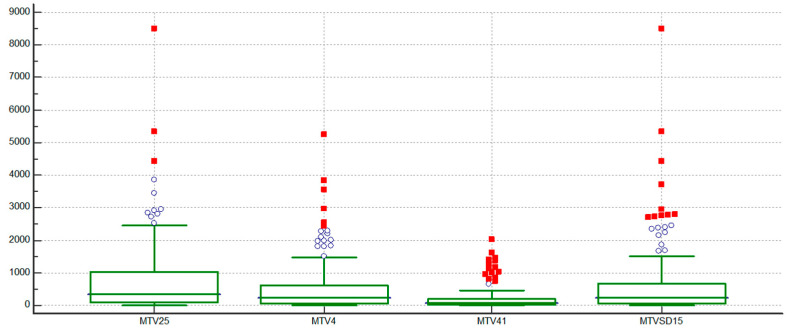
A boxplot diagram showing distributions of the metabolic tumor volumes (MTVs). Collected data: The median and interquartile range (box), the mean (line within the box), outliers (circles), and extreme outliers beyond 3 times the interquartile range (squares). MTV25, MTV4, MTV41, MTVSD15: MTVs calculated using a SUV threshold of ≥2.5 g/mL, ≥4.0 g/mL, >41% SUVmax, and ≥1.5 × liver SUVmean + 2 standard deviations, respectively.

Distribution of MTV based on different thresholding methods is shown in Figure 1.

In the regression analyses of pairwise comparisons between MTV25, MTV4, MTV41, and MTVSD15, the strongest correlations were observed between MTV4 and MTVSD15, MTV25 and MTV4, and MTV25 and MTVSD15, with R^2^ values consistently high at 0.96, 0.95, and 0.95 (all *p* < 0.0001), respectively. In contrast, all comparisons involving MTV41 showed moderate correlations, with R^2^ values ranging from 0.70 to 0.76 (all *p* < 0.0001).

When comparing the regression-derived equivalents of MTV41, calculated using linear regression equations based on MTV25, MTV4, and MTVSD15 (designated as MTV41′ (MTV25), MTV41 (MTV4), and MTV41′ (MTVSD15), respectively), very strong correlations were observed between MTV41′ (MTV4) and both MTV25 and MTVSD15, with R^2^ values of 0.95 and 0.96, respectively (both *p* < 0.0001), while the comparison involving MTV41 showed only moderate correlation, with an R^2^ of 0.79 (*p* < 0.0001) (Table 3).

A Bland–Altman pairwise comparison of MTVs across the four PET threshold methods demonstrated that the absolute SUV thresholds (MTV25, MTV4, and MTVSD15) had narrower limits of agreement and thus better overall agreement compared to the relative threshold MTV41. The observed systematic bias between methods was statistically significant (*p* < 0.0001) for all pairs. When comparing the original MTV41 to the regression-derived variables MTV41′ (MTV25), MTV41′ (MTV4), and MTV41′ (MTVSD15), no significant systematic bias was observed, with all *p*-values approaching 1. Furthermore, these regression-derived MTV41′ variables exhibited the narrowest limits of agreement across all pairs studied (Table 4, and Supplement S1).

In a Spearman rank correlation analysis, a robust correlation was evident among the MTVs and TLGs derived from all segmentation methods, indicating a strong association between these variables (Table 5). Additionally, there was an anticipated tendency for correlation between IPI and stage, as both contributed to the calculation of IPI. A notable correlation (ρ = 0.75) was also observed between SUVmax and the LLR, as expected, given that LLR represents SUVmax normalized to background uptake in the liver; however SUVmax did not correlate to any of the PET volumetric parameters.

For the overall cohort, several baseline PET-derived volumetric parameters and IPI demonstrated significant prognostic value for PFS2. In ROC analysis, with IPI showed the strongest discriminatory power (AUC = 0.733), followed by MTV25 (AUC = 0.674), MTV41 (AUC = 0.673), and several TLG measures (AUC range: 0.647–0.669), all with statistically significant *p*-values. In contrast, SUVmax showed no predictive value (AUC = 0.504, *p* = 0.95), indicating no discriminative power for PFS2 (Supplement S2, Table S2.1). Kaplan–Meier analysis further supported these findings, as all four MTV methods (MTV25, MTV4, MTV41, MTVSD15) were significantly associated with PFS2 (*p*-values ranging from 0.0005 to 0.0015), with HRs between 3.39 and 4.36. IPI showed the strongest prognostic impact (HR = 5.57, *p* < 0.0001), followed closely by LLR (HR = 5.52, *p* = 0.0042) (Supplement S2, Table S2.2).

The ROC analysis of all MTV and TLG thresholding methods for OS3 demonstrated comparable curve progression, with AUC values ranging from 0.64 to 0.69 (*p* = 0.002–0.03) (Table 6). While the ROC curve for the IPI showed superior performance, the ROC analysis for the LLR and SUVmax underperformed and did not reach statistical significance (Table 6 and Supplement S3).

Pairwise comparisons of the ROC curves revealed significant differences between all MTV variables and IPI compared to LLR (*p* range = <0.001–0.025), and between MTVSD15 and IPI (*p* = 0.03). Other comparisons between ROC curve pairs did not show significant differences.

A separate ROC analysis was performed for the stage 4 patient subgroup (n = 84), where only IPI (AUC = 0.70, *p* = 0.001) and MTV41 (AUC = 0.64, *p* = 0.04) remained statistically significant. IPI continued to be the strongest predictor in both patient groups, although its optimal threshold shifted from >3 in the overall cohort to >4 in the stage 4 subgroup. IPI also demonstrated a higher AUC (0.76 vs. 0.70) and sensitivity (64% vs. 42%) in the overall population, whereas specificity was greater in stage 4 patients (88% vs. 74%) (Table 6 and Supplement S4).

For both OS and PFS, the median survival was not reached (95% confidence interval [95% CI] not reached); the median follow-up was 54.3 months (OS: 95% CI, 49.2–59.7; PFS: 95% CI, 49.2–60.3 months). The median follow-up time to calculate the Kaplan–Meyer curves for OS was 46.8 months (range: 2.0–85.4 months; interquartile range (IQR): 29.4–63.0 months). A total of 79 patients (56%) achieved CR after first-line treatment; 40 patients (29%) achieved a partial response followed by curative radiotherapy; 21 patients (15%) progressed during initial treatment, with 9 (6%) dying during treatment; and an additional 25 patients (18%) died during follow-up (median: 17.2 months; IQR: 12.6 months–29.4 months).

Kaplan–Meier analysis of OS of patients dichotomized with MTV25, MTV4, MTV41, MTVSD15, LLR, SUVmax, and IPI, using ROCs-derived optimal threshold values of >477 mL, >171 mL, >150 mL, >176 mL, >20.6, ≤25.94, and >3, respectively. A significantly poorer OS3 was observed in patients with higher IPI score (*p* = 0.0001, hazard ratio [HR] = 4.18; 95% CI, 1.87–9.39), higher values of LLR (0.01, 2.82, 0.92–8.67), MTV25 (0.002, 3.34, 1.56–7.13), MTV4 (0.003, 3.65, 1.74–7.65), MTV41 (0.001, 3.30, 1.49–7.31), and MTVSD15 (0.003, 3.53, 1.68–7.40, respectively), while SUVmax did not reach significance (Table 7 and Supplement S3). In the stage 4 subgroup, IPI (*p* = 0.0025, HR = 3.14) remained the most robust prognostic factor, with the highest AUC (0.70) and specificity (88%). Among PET metrics, only MTV25 and MTV41 remained significant predictors (*p* = 0.014 and 0.041, respectively) (Table 7 and Supplement S4).

In the univariate Cox analysis on log2 transformed data of different threshold methods, all MTV metrics were significantly associated with OS3. HRs ranged from 1.17 to 1.27, with significant *p*-values (from 0.013 to 0.038), indicating that MTV measures have prognostic value regardless of the thresholding method used, when considered individually. MTV41 performed slightly superior, exhibiting the highest HR and the greatest statistical significance (*p* = 0.003) (Table 8). LLR yielded a non-significant result (HR = 1.00, *p* = 0.99). IPI displayed a strong prognostic impact, with an HR of 1.95 (*p* < 0.0001), confirming its established role as a key prognostic factor in DLBCL. When adjusting for IPI, the significance of all MTV metrics was lost, with HR values approaching 1 and *p*-values becoming non-significant (0.85–0.98), indicating that these MTV metrics do not provide additional independent prognostic information beyond IPI. LLR remained non-significant in the multivariate analysis. IPI maintained a strong and highly significant association with the outcome in all models (HR from 1.82 to 2.06, *p*-values ranging from <0.0001 to 0.0009), indicating its robustness as a prognostic marker.

Each model incorporating IPI and a PET metric resulted in a chi-squared value of approximately 20 to 21, with highly significant *p*-values (*p* < 0.00001), suggesting that the models overall are strongly predictive of the outcome.

## 4. Discussion

Pretreatment MTV measured by PET/CT offers valuable prognostic information on DLBCL, which often outperforms standard international prognostic scores [15,16,28]. Despite its potential, the lack of standardized MTV measurement protocols limits its use in clinical trials and routine practice. In this study, greater MTV tumor burdens, regardless of the PET segmentation method used, were significantly associated with survival and PFS2 in the overall patient cohort, confirming the results of several previous studies [11,12,13,14,28,29,30], whereas in the stage 4 patient subgroup, significance persisted only for MTV25 and MTV41. The strong similarities observed between PFS2 and OS3 in our analysis likely reflect the underlying biology and clinical course of aggressive lymphomas such as DLBCL, where early progression often leads to poor long-term outcomes. Both endpoints were significantly associated with key PET-derived volumetric parameters (e.g., MTV25, MTV41) and IPI, suggesting that the disease burden measured at baseline and adverse clinical features contribute not only to earlier relapse but also to increased long-term mortality risk. PFS2 and OS3 seem to be interrelated: Patients who progress within the first two years frequently receive intensive salvage therapy or experience treatment resistance, both of which are linked to reduced OS. Thus, imaging and clinical biomarkers that effectively predict early progression inherently carry prognostic value for long-term survival, explaining the concordant patterns seen across ROC and Kaplan–Meier analyses for PFS2 and OS3. When considering the multivariate analysis, none of the measures evaluated in our study stood out as performing better than the others, as they lost significance when adjusted for IPI. However, considering univariate analysis, MTV41 showed the best performance with the highest HR and the most significant *p*-value, confirming the recommendations of the EANM for the SUV threshold method of tumor burden evaluation and the results of another manual segmentation study [23,31]. Nevertheless, the differences among the various PET metrics were minimal and are unlikely to have a significant impact on clinical reporting [32]. We found that the >41% SUVmax threshold method did not result in previously reported underestimation of the disease burden in large pathologic lesions with high FDG uptake [33], which would be significant for prognostication, despite inclusion of patients with very high SUVs to our study (median SUVmax 26.8, range exceeding 59). On the contrary, in our analysis, the maintained significance of MTV41 in stage 4 patients highlighted its potential as a robust prognostic indicator, even in advanced disease stages. The higher median MTV41 in stage 4 (85.5 mL vs. 64.5 mL in overall cohort) and wider confidence intervals (61.0–159.5 mL vs. 41.2–88.3 mL) suggest that this method effectively captures the increased tumor burden and metabolic activity in advanced cancer, which could be helpful for treatment decisions. Contrary to a report by Eude et al. [34], LLR did not show a significant result (*p* = 0.99) in our study, suggesting that this biomarker may not independently predict the outcome.

Variations in results across studies may be due to differences in the handling of data, such as whether or not log transformations were applied to normalize non-normal distributions. Some studies may have used normalized data, while others did not, leading to inconsistencies in HR comparisons. Additionally, differences in the stage of included patients, variations in histology, the presence of secondary indolent lymphoma, or the diversity in treatment regimens can significantly impact outcomes and also contribute to heterogeneity between studies.

Our study, consistent with previous reports, found that SUVmax was not significantly associated with survival [35]. Although SUVmax is a commonly used parameter in routine clinical reporting to confirm and quantify tumor malignancy, it has several limitations. These include its inability to reflect the total tumor burden and its susceptibility to variations in uptake time, scan duration, acquisition and reconstruction protocols, blood glucose levels, and partial volume effect [36].

While none of the mean differences between the original MTV41 and the regression-derived MTV41′ values were significantly different from zero, the wide limits of agreement across all pairs of original and regression-derived values suggest substantial variability. Therefore, the results of our study do not support the use of regression-derived MTV41′ values based on simpler fixed thresholding methods as reliable substitutes for the original MTV41 in clinical and research applications.

Interestingly, despite several reports reporting PET tumor burden biomarkers as superior compared to IPI for prediction of outcome in DLBCL patients [15,18,37,38], IPI remained the dominant prognostic factor for DLBCL in our dataset of 140 patients, including 84 stage 4 patients. This finding underscores the continued relevance of IPI as a robust predictor of outcomes in this context [30,39]. IPI integrates five clinical parameters—age, LDH, ECOG performance status, Ann Arbor stage, and extranodal sites [40]—offering a comprehensive assessment of both patient and disease characteristics. Likewise, the recently proposed Integrated Metabolic Prognostic Index (IMPI), which combines total MTV with age and stage as continuous variables [17], has not proven sufficiently robust in routine practice and has not surpassed the predictive power of IPI [41].

Several factors may explain IPI’s dominance in our study. IPI’s inclusion of patient-related factors (age, performance status) and disease-related factors (stage, LDH, extranodal sites) captures a broader prognostic landscape than PET-derived variables, which focus solely on tumor metabolism. For instance, elderly patients with poor performance status may have had worse outcomes regardless of metabolic tumor burden, an aspect that the IPI effectively addresses. Our cohort’s high proportion of stage 4 patients (84/140) likely amplified the prognostic weight of IPI’s stage and extranodal components. In contrast, while PET-derived variables offer valuable insights into tumor biology, their utility in our dataset was limited, likely due to technical [9] and cohort-specific factors.

Several authors recommended the use of fixed SUV4 threshold as the most optimal segmentation method for prognostication of baseline disease burden in DLBCL patients. However, these data are based on a small number of patients included in the study [42,43] or on automatic image analysis [33], and therefore cannot be directly compared. Based on our analysis, a segmentation threshold of >41% SUVmax warrants further investigation. Nevertheless, since all four threshold methods demonstrated comparable prognostic efficacy, the choice of the optimal method may depend on factors such as performance success, ease of use, time efficiency, and the level of user involvement in tumor burden assessment, as previously noted by Barrington et al. [33].

In DLBCL, the use of automatic segmentation for PET/CT images is becoming increasingly common. AI methods offer significant advantages over manual segmentation, such as improved time efficiency, reduced inter- and intra-observer variability, and the ability to process large datasets consistently, making them a promising tool for future clinical applications [33,44,45]. Nevertheless, AI-based segmentation of PET images has notable drawbacks compared to manual segmentation, including potential inaccuracies in cases of heterogeneous nodal and extranodal masses, a lack of adaptability to tumor presentations in the areas of physiological uptake such as the myocardium, bladder, kidneys, bowel, and brain [46], and the need for extensive, high-quality annotated datasets for training. The performance of AI-based delineation methods in lymphoma is lower compared to segmentation of other cancers [47].

In our study, which included a predominantly older population (69% >60 years, median age 66 years) with advanced-stage disease (stage III/IV: 74%), the optimal MTV41 threshold for prognostic discrimination was 150 mL, notably lower than those reported in previous studies. Sasanelli et al., with a younger cohort (median age 56 years, only 31% >60 years), reported a much higher MTV41 threshold of 550 mL, despite a similarly high proportion of advanced-stage disease (82%) [11]. Toledano et al. also used a 41% SUVmax threshold and identified a MTV cutoff of 261 mL in a population where 68.4% of patients were over 60 years, supporting the idea that older patients have worse tolerance for higher tumor burden [29]. Capobianco et al. in the REMARC study (patients aged 60–80 years, 91% stage III/IV), reported a manual segmentation threshold of 223 mL and, using the AI-assisted PARS method, a remarkably similar threshold of 148 mL [48]. These findings align with the hypothesis proposed by Toledano et al. that the prognostically relevant MTV threshold tends to decrease with increasing patient age, as older patients appear less capable of tolerating high tumor volumes. Our results are consistent with this trend and underscore the importance of considering age and disease stage when interpreting MTV thresholds for prognostic stratification. However, it is important to emphasize that comparing absolute MTV threshold values across studies is not advisable due to numerous sources of variability. Differences in PET acquisition protocols, scanner technology, reconstruction algorithms, and image processing methodologies all affect SUV measurements and MTV calculations [49]. Therefore, threshold values are study-specific and should be interpreted within the context of each study rather than as universally applicable cutoffs.

A persistent challenge in lymphoma segmentation is distinguishing diffuse splenic FDG uptake caused by reactive, nonmalignant processes from true lymphoma involvement [9,46]. In theory, misclassifying benign splenic uptake could increase MTV and impair prognostic accuracy; however, MTV’s predictive power is primarily driven by the total tumor burden across all sites, rather than uptake in any single organ, like the spleen. Indeed, isolated splenic involvement in FDG PET/CT has not proven an adverse prognostic factor for OS [50,51], indicating that low-activity benign splenic uptake contributes negligibly to whole-body MTV and overall outcome predictions. Our findings corroborate this: a possible unintentional inclusion of minimal nonpathological splenic uptake in MTV25 did not reduce its prognostic value.

Several groups have proposed criteria to identify true splenic infiltration. Kozuki et al. suggested an absolute spleen SUVmax cutoff of 3.48 (*p* = 0.000; sensitivity 82%, specificity 90%) [50], while Hu et al. reported a significant difference in spleen SUVmax between B-cell lymphoma and benign spleen findings (*p* = 0.027), and determined an optimal spleen to liver SUVmax ratio of 2.42 (*p* = 0.001; sensitivity 62.5%; specificity 100%) for distinguishing lymphoma from benign uptake [52]. Based on normal physiological liver uptake (SUVmax 2.81 ± 0.46; SUVmean 2.26 ± 0.38) [53], excluding splenic uptake below approximately 1.5 × liver SUVmax provides a conservative threshold to omit nonpathological uptake from MTV calculations.

Moreover, central necrosis within malignant lesions presents an additional challenge. Because necrotic regions lack significant FDG uptake, they are often omitted from MTV calculations, potentially underestimating true tumor burden [35]. While PET/CT primarily captures increased metabolic activity, accounting for necrosis could provide more comprehensive insights into the tumor’s aggressiveness, prognosis, and treatment response [54,55,56], as the presence of necrotic areas is itself an independent adverse prognostic factor, reflecting hypoxia and treatment resistance [57,58]. Future lymphoma PET biomarker studies should consider quantifying hypoxic/necrotic regions using hypoxia-specific radiotracers, like ^18^F-FMISO, which may serve as novel independent prognostic markers.

Achieving reproducibility of segmentation data across different clinical settings and research sites is critical for standardizing outcomes in DLBCL. To improve reproducibility, efforts should focus on developing and adhering to standardized segmentation protocols [46], which may include harmonized acquisition and reconstruction parameters, as well as automated tools with built-in error-correction algorithms that account for variations in anatomical and physiological contexts. Another step could be the integration of hybrid approaches, combining automated segmentation with expert manual corrections, to ensure that key regions, such as necrotic areas and those near high-uptake organs, are accurately assessed.

Our study has several limitations. First, the retrospective design relies on pre-existing data, which may introduce inconsistencies and limit the representativeness of the study population, introduce biases and limit control over confounding factors, and affect the representativeness of the data. Furthermore, the analysis may be subjected to bias due to the use of a single reviewer for PET image interpretation, however limiting potential interobserver variability that could influence the findings. Additionally, we did not employ an advanced automatic segmentation protocol, as this method is still under development within our research group. The lack of automation is particularly important because it limits the comparability of our results with those of other research teams using automated methods. Nevertheless, similar to manual segmentation techniques, no preferred automated segmentation method has been established for patients with NHL [44]. We acknowledge that the prognostic value of MTV-based tumor burden metrics should not be directly compared across studies. Absolute MTV thresholds are highly sensitive to variations in PET scanner technology, reconstruction algorithms, and correction methods [59], as well as to differences in patient populations, tumor types, and metabolic characteristics. These factors introduce significant variability in MTV measurements, limiting cross study comparability. Future meta analyses should account for this variability. For clinical implementation, institutions may need to develop and validate their own thresholds tailored to local imaging protocols and patient cohorts.

In summary, while none of the MTV metrics evaluated in our study stood out as performing better than the others in the entire cohort, MTV25 and MTV41 remained significant in the stage 4 patient subgroup. Based solely on the univariate analysis, MTV41 showed the best performance with the highest HR and the most significant *p*-value. However, the regression-derived MTV41′ values, based on simpler fixed thresholding methods, do not serve as reliable substitutes for the original MTV41. The PET metrics’ prognostic value was diminished when adjusted for IPI, which remained the dominant prognostic factor in DLBCL. This suggests that IPI continues to be the most robust predictor of outcomes in this context.

## 5. Conclusions

While MTV metrics derived from PET/CT provide significant univariate prognostic information, there is little difference in selection of threshold of abnormal uptake, and their added value diminishes when combined with IPI, which remains the most reliable predictor of survival in DLBCL.

## Figures and Tables

**Table 1:** Clinical characteristics of patients.

Age at diagnosis [years]: median (range)	66 (20–80)
Gender, female/male, n (%)	59 (42%)/81 (58%)
IPI score, n (%)	
IPI Low-risk group (LR): 29 (21%)	
IPI Low intermediate-risk group (LIR): 30 (21%)	
IPI High intermediate-risk group (HIR): 33 (24%)	
IPI High-risk group (HR): 48–(34%)	
Stage at diagnosis, n (%)	II: 36 (26%)
	III: 20 (14%)
	IV: 84 (60%)
Chemotherapy regimen, n (%)	R-CHOP: 108 (77%)
	REPOCH: 11 (8%)
	RCOEP: 6 (4%)
	Reduced R-CHOP (mini-RCHOP; 80%, 75%, 50%): 6 (4%)
	RACVBP: 5 (4%)
	Other: 6 (4%)
Extranodal sites: 0, n (%)	24 (17%)
Extranodal sites: 1, n (%)	43 (31%)
Extranodal sites more than 1, no. (%)	73 (42%)
Serum LDH elevated	76 (54%)

IPI: International Prognostic Index; LDH: lactate dehydrogenase.

**Table 2:** Median values, 95% confidence intervals, ranges, and interquartile range for PET metrics and International Prognostic Index score.

	Median	95% CI of Median	Range	IQR
SUVmax	26.8	24.3–29.0	3.6–59.6	20.3–33.9
MTV25 [mL]	331.1	208.3–482.0	2.3–8498.1	90.1–1038.3
MTV4 [mL]	224.9	141.3–344.6	0–5243	54.5–626.7
MTV41 [mL]	64.5	41.2–88.3	0.4–2031.0	17.1–196.7
MTVSD15 [mL]	225.3	153.2–362.3	0.2–8498.1	53.4–680.4
LLR	12.9	11.9–13.8	2.0–35.5	9.7–17.0
TLG25	2618.6	1813.1–4258.6	6.7–43106.2	591.4–8268.4
TLG4	2170.5	1454.0–3662.8	0–37128.9	488.8–7413.9
TLG41	1209.3	651.9–1541.6	5.3–27069.3	229.4–3566.6
TLGSD15	2286.9	1478.8–3605.2	0.8–43106.2	494.1–7436.4
IPI	3	2–3	0–5	2–4

95% CI, 95% confidence intervals; IQR, interquartile range; MTV25, MTV4, MTV41, MTVSD15, TLG25, TLG4, TLG41, TLGSD15: MTVs and TLGs calculated using a SUV threshold of ≥2.5, ≥4.0, >41%SUVmax, and ≥1.5 × liver SUVmean + 2 standard deviations, respectively. LLR, lesion-to-liver ratio; IPI, International Prognostic Index score.

**Table 3:** Regression analyses of pairwise comparisons between various metabolic tumor volumes (MTVs).

Variables Compared	R^2^ Value	*p*-Value	Intercept	Intercept SE	Slope	Slope SE
MTV4/MTV25	0.95	*p* < 0.0001	35.49	27.64	1.35	0.03
MTV4/MTV41	0.79	*p* < 0.0001	−9.23	17.45	0.38	0.02
MTV4/MTVSD15	0.96	*p* < 0.0001	81.24	16.09	0.73	0.01
MTV41/MTV25	0.70	*p* < 0.0001	−0.59	20.80	0.26	0.01
MTV41/MTVSD15	0.76	*p* < 0.0001	22.27	18.02	0.28	0.01
MTV25/MTVSD15	0.95	*p* < 0.0001	133.76	27.29	1.00	0.02
MTV25/MTV41′ (MTV4)	0.95	*p* < 0.0001	63.89	27.37	3.56	0.07
MTV41/MTV41′ (MTV4)	0.79	*p* < 0.0001	−1.24	17.25	1.01	0.04
MTVSD15/MTV41′ (MTV4)	0.96	*p* < 0.0001	−55.16	22.18	3.49	0.06

MTV25, MTV4, MTV41, MTVSD15, and regression-derived MTV41′ variable [MTV41′ (MTV4)], calculated using SUV thresholds of ≥2.5 g/mL, ≥4.0 g/mL, >41% of SUVmax, and ≥1.5 × liver SUVmean + 2 standard deviations, respectively. The regression-derived variable MTV41′ (MTV4) was calculated based on MTV4. SE: standard error.

**Table 4:** Bland–Altman analyses of pairwise comparisons between various metabolic tumor volumes (MTVs).

Variables Compared	*p*-Value	Mean Difference	Lower Limits of Agreement	Upper Limits of Agreement
MTV4/MTV25	*p* < 0.0001	239	−554	1032
MTV4/MTV41	*p* < 0.0001	367	−722	1456
MTV4/MTVSD15	*p* < 0.0001	−105	−799	589
MTV41/MTV25	*p* < 0.0001	606	−1163	2376
MTV41/MTVSD15	*p* < 0.0001	−472	−2145	1202
MTV25/MTVSD15	*p* < 0.0001	135	−408	677
MTV41/MTV41′ (MTV25)	*p* = 0.99	0	−396	395
MTV41/MTV41′ (MTV4)	*p* = 0.97	−1	−334	333
MTV41/MTV41′ (MTVSD15)	*p* = 0.92	2	−356	360

MTV25, MTV4, MTV41, MTVSD15, and regression-derived MTV41′ variables [MTV41′ (MTV25), MTV41′ (MTV4), and MTV41′ (MTVSD15)]. MTVs were calculated using SUV thresholds of ≥2.5 g/mL, ≥4.0 g/mL, >41% of SUVmax, and ≥1.5 × liver SUVmean + 2 standard deviations, respectively. The regression-derived MTV41′ variables were calculated from MTV25, MTV4, and MTVSD15, respectively.

**Table 5:** Spearman rank correlations.

	MTV25	TLG25	SUVmax	LLR	MTV4	TLG4	MTV41	TLG41	MTVSD15	TLGSD15	MIB1	Stage	IPI	LDH
MTV25		**0.93**	0.04	**0.46**	**0.98**	**0.9**	**0.8**	**0.74**	**0.96**	**0.9**	−0.04	**0.37**	**0.51**	**0.64**
TLG25	**0.93**		**0.17**	**0.61**	**0.96**	**1**	**0.87**	**0.91**	**0.94**	**0.99**	−0.02	**0.36**	**0.51**	**0.61**
SUVmax	0.04	**0.17**		**0.75**	0.07	**0.19**	−0.05	0.13	0.04	**0.18**	0.11	0.06	0.09	−0.03
LLR	**0.46**	**0.61**	**0.75**		**0.52**	**0.63**	**0.41**	**0.56**	**0.52**	**0.63**	0.08	**0.22**	**0.3**	**0.3**
MTV4	**0.98**	**0.96**	0.07	**0.52**		**0.95**	**0.85**	**0.82**	**0.99**	**0.96**	−0.04	**0.35**	**0.49**	**0.63**
TLG4	**0.9**	**1**	0.19	**0.63**	**0.95**		**0.87**	**0.93**	**0.92**	**1**	−0.02	**0.34**	**0.49**	**0.59**
MTV41	**0.8**	**0.87**	−0.05	**0.41**	**0.85**	**0.87**		**0.93**	**0.83**	**0.87**	−0.08	**0.29**	**0.45**	**0.66**
TLG41	**0.74**	**0.91**	0.13	**0.56**	**0.82**	**0.93**	**0.93**		**0.79**	**0.92**	−0.03	**0.29**	**0.45**	**0.58**
MTVSD15	**0.96**	**0.94**	0.04	**0.52**	**0.99**	**0.92**	**0.83**	**0.79**		**0.94**	−0.03	**0.33**	**0.47**	**0.63**
TLGSD15	**0.9**	**0.99**	0.18	**0.63**	**0.96**	**1**	**0.87**	**0.92**	**0.94**		−0.02	**0.34**	**0.49**	**0.59**
MIB1	−0.04	−0.02	0.11	0.08	−0.04	−0.02	−0.08	−0.03	−0.03	−0.02		−0.01	−0.03	0
Stage	**0.37**	**0.36**	0.06	**0.22**	**0.35**	**0.34**	**0.29**	**0.29**	**0.33**	**0.34**	−0.01		**0.78**	**0.32**
IPI	**0.51**	**0.51**	0.09	**0.3**	**0.49**	**0.49**	**0.45**	**0.45**	**0.47**	**0.49**	−0.03	**0.78**		**0.47**
LDH	**0.64**	**0.61**	−0.03	**0.3**	**0.63**	**0.59**	**0.66**	**0.58**	**0.63**	**0.59**	0	**0.32**	**0.47**	

ρ Spearman rank correlation coefficients of PET and clinical variables. A linear three-color gradient map indicates a strong positive correlation (dark green), weak or no correlation (yellow), and strong negative correlation (red). In this dataset, correlations ranged from no correlation to strong positive correlation. Since no meaningful negative correlations were present, red does not appear in the figure. Lighter green shades represent weaker positive correlations, while darker green indicates stronger positive correlations. Statistically significant is represented in bold. MTV25, MTV4, MTV41, MTVSD15, TLG25, TLG4, TLG41, TLGSD15: MTVs and TLGs calculated using a SUV threshold of ≥2.5 g/mL, ≥4.0 g/mL, >41% SUVmax, and ≥1.5 × liver SUVmean + 2 standard deviations, respectively. LLR, lesion-to-liver ratio; MIB1, MIB-1 immunohistochemical proliferation index; LDH, lactate dehydrogenase; IPI, International Prognostic Index score.

**Table 6:** Receiver-operating characteristics analysis of prognostic factors for three-year overall survival.

ALL Patients, n = 140							Stage 4 Patient Subgroup, n = 84					
	AUC	AUC 95% CI	Sensitivity [%]	Specificity [%]	*p*-Value	Optimal Threshold Value for Predicting OS3	AUC	AUC 95% CI	Sensitivity [%]	Specificity [%]	*p*-Value	Optimal Threshold Value for Predicting OS3
SUVmax	0.56	0.47–0.66	61	57	0.32	≤25.94	0.54	0.42–0.66	54	63	0.57	≤25.9
MTV25	0.67	0.57–0.75	68	62	0.008 *	<478 mL	0.60	0.48–0.72	75	48	0.16	>478 mL
MTV4	0.65	0.56–0.74	79	49	0.01 *	<171 mL	0.60	0.48–0.71	75	46	0.17	>256 mL
MTV41	0.69	0.59–0.77	61	72	0.002 *	<150 mL	0.64	0.52–0.75	83	48	0.04 *	>60.3 mL
MTVSD15	0.64	0.54–0.73	79	48	0.03 *	<176 mL	0.58	0.45–0.69	79	40	0.30	>199 mL
LLR	0.51	0.42–0.61	29	91	0.85	<20.7	0.51	0.39–0.63	29	85	0.93	≤8.2
TLG25	0.66	0.57–0.75	79	55	0.01 *	<2562.64	0.62	0.49–0.73	83	44	0.11	>2563
TLG4	0.64	0.54–0.73	71	56	0.03 *	<2200.48	0.60	0.47–0.71	75	46	0.19	>2200
TLG41	0.66	0.56–0.74	32	94	0.01 *	<7033.83	0.63	0.51–0.74	88	38	0.071	>479
TLGSD15	0.63	0.54–0.72	75	54	0.04 *	<2278.32	0.59	0.47–0.70	83	38	0.22	>1525
IPI	0.76	0.67–0.83	64	74	<0.0001 *	>3	0.70	0.58–0.80	42	88	0.001 *	>4

AUC, Area under the receiver-operating characteristic curve; 95% CI, 95% confidence intervals; MTV25, MTV4, MTV41, MTVSD15, TLG25, TLG4, TLG41, TLGSD15: MTVs and TLGs calculated using a SUV threshold of ≥2.5 g/mL, ≥4.0 g/mL, >41% SUVmax, and ≥1.5 × liver SUVmean + 2 standard deviations, respectively; LLR, lesion-to-liver ratio; IPI, International Prognostic Index score. * indicates statistical significance at *p* < 0.05. Sensitivity and specificity are reported at optimal cutoffs determined by Youden’s index.

**Table 7:** Kaplan–Meier analysis of OS3 dichotomized by MTV25, MTV4, MTV41, MTVSD15, IPI, SUVmax, and LLR, using ROC-derived optimal thresholds for all patients and the stage 4 patient subgroup.

	ALL Patients, n = 140			Stage 4 Patient Subgroup, n = 84		
	*p*-Value	HR	95% CI	*p*-Value	HR	95% CI
MTV25	0.002 *	3.34	1.56–7.13	0.014 *	2.97	1.37–6.41
MTV4	0.003 *	3.65	1.74–7.65	0.06	2.23	1.03–4.82
MTV41	0.001 *	3.30	1.49–7.31	0.041 *	2.50	1.15–5.43
MTVSD15	0.003 *	3.53	1.68–7.40	0.13	1.98	0.89–4.39
IPI	0.0001 *	4.18	1.87–9.39	0.0025 *	3.14	1.12–8.83
SUVmax	0.15	1.67	0.83–3.33	0.51	1.30	0.60–2.82
LLR	0.01 *	2.82	0.92–8.67	0.10	0.50	0.17–1.44

OS3, three-year overall survival; IPI, International Prognostic Index score; LLR, lesion–to–liver ratio; MTV25, MTV4, MTV41, and MTVSD15: MTVs calculated using a SUV threshold of ≥2.5 g/mL, ≥4.0 g/mL, >41% SUVmax, and ≥1.5 × liver SUVmean + 2 standard deviations, respectively. HR, hazard ratio; 95% CI, 95% confidence interval. * indicates statistical significance at *p* < 0.05.

**Table 8:** Univariate and multivariate Cox regressions of three-year overall survival on log2-transformed data of different thresholding methods MTVs compared with IPI.

	Univariate Analysis			Multivariate Analysis with IPI							
	HR	95% CI	*p*-Value	HR	95% CI	*p*-Value	HR (IPI)	95% CI (IPI)	*p*-Value (IPI)	Chi-Squared	*p*-Value
log2 MTV25	1.25	1.05–1.48	0.013 *	1.02	0.85–1.22	0.85	1.92	1.34–2.76	0.0004 *	20.25	<0.0001
log2 MTV4	1.19	1.01–1.39	0.037 *	0.99	0.85–1.17	0.98	1.95	1.37–2.76	0.0002 *	19.95	<0.0001
log2 MTV41	1.27	1.08–1.48	0.003 *	1.08	0.93–1.26	0.30	1.82	1.28–2.58	0.0009 *	21.35	<0.0001
log2 MTVSD15	1.17	1.01–1.36	0.038 *	0.99	0.86–1.15	0.96	1.96	1.38–2.78	0.0002 *	20.21	<0.0001
log2 LLR	1.00	0.62–1.62	0.99	0.72	0.45–1.17	0.19	2.06	1.48–2.88	<0.0001 *	21.84	<0.0001
IPI	1.95	1.42–2.69	<0.0001 *								

MTV25, MTV4, MTV41, and MTVSD15: MTVs calculated using a SUV threshold of ≥2.5 g/mL, ≥4.0 g/mL, >41% SUVmax, and ≥1.5 × liver SUVmean + 2 standard deviations, respectively. LLR, lesion-to-liver ratio; IPI, International Prognostic Index score. * indicates statistical significance at *p* < 0.05.

## Data Availability

The raw data supporting the conclusions of this article will be made available by the authors on request.

## Supplementary Materials

The following supporting information can be downloaded at: https://www.mdpi.com/article/10.3390/curroncol32060356/s1; Supplement S1. Bland–Altman pairwise comparison of MTVs across the four PET threshold methods; Supplement S2. Receiver-operating characteristic curves and Kaplan–Meier curves of PFS in all patients; Supplement S3. Receiver-operating characteristic curves and Kaplan–Meier curves of OS in all patients; Supplement S4. Receiver-operating characteristic curves and Kaplan–Meier curves of OS in the stage 4 patient subgroup.
