# Supplementary material for: Prognostic Value of Multiple Manual Segmentation Methods for Diffuse Large B-Cell Lymphoma with 18F-FDG PET/CT
Source: Curr Oncol. 2025 Jun 16;32(6):356. doi: 10.3390/curroncol32060356 (PMC12191845; doi:10.3390/curroncol32060356)
Supplement: Supplementary file 1 [file curroncol-32-00356-s001.zip › Supplement S1.pdf]

Supplement S1. Bland–Altman pairwise comparison of MTVs across the four PET threshold methods.

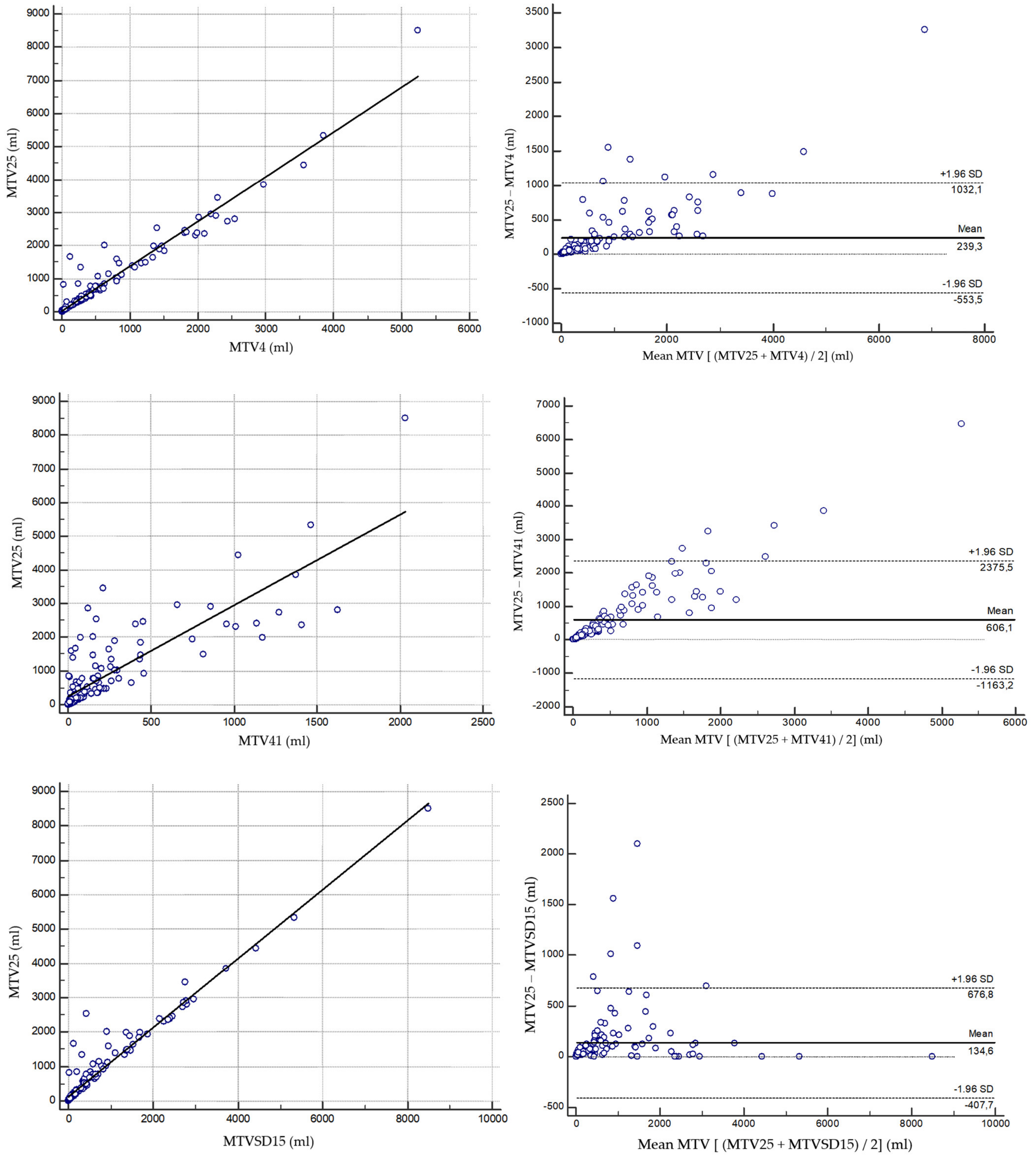

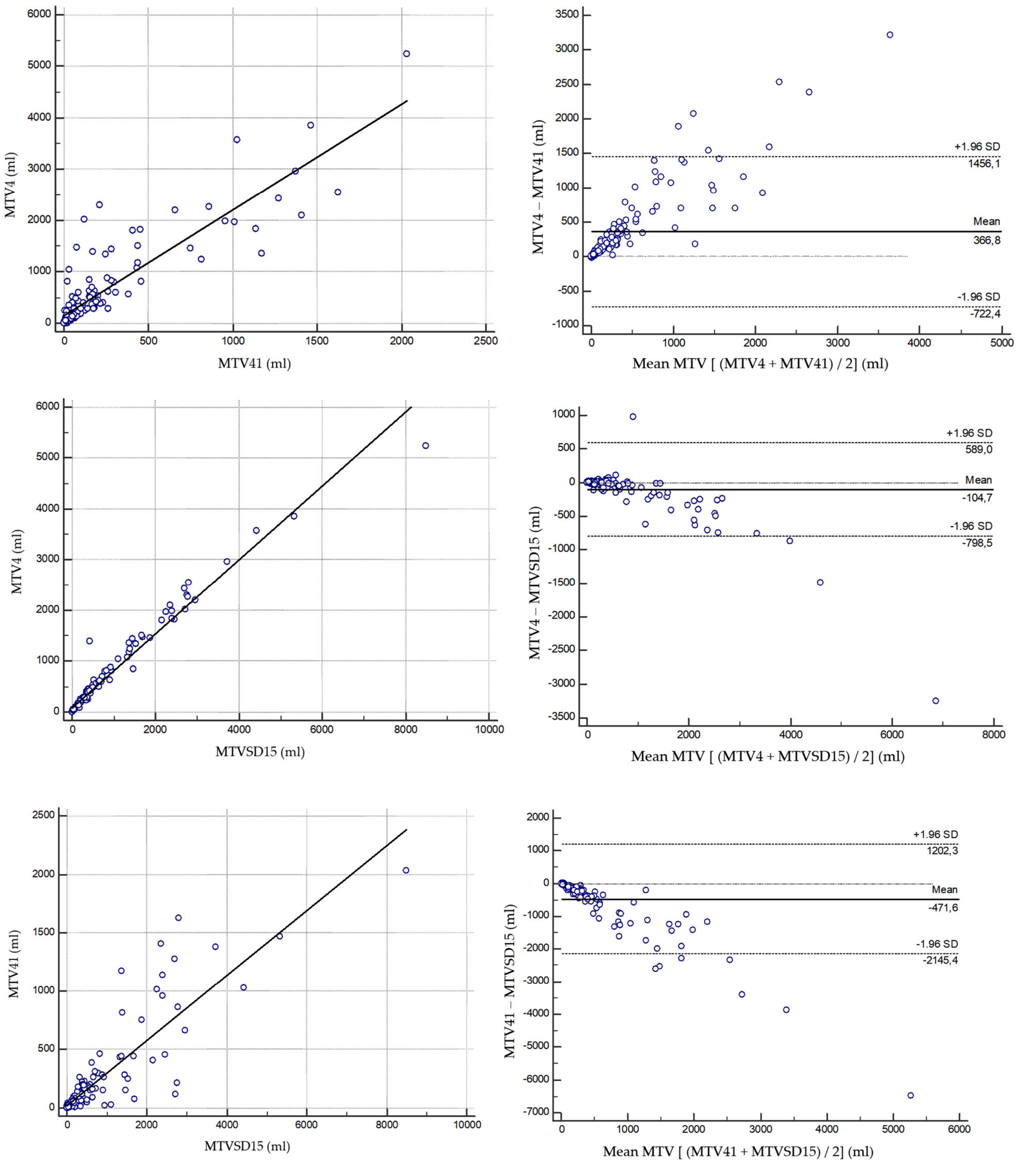

Figure S1.1. Regression analysis scatter plots (left column) and Bland−Altman plot analysis with mean and standard deviation (SD) lines (right column) of metabolic tumor volumes (MTV) across four PET threshold methods: MTV25, MTV4, MTV41, and MTVSD15, calculated using a SUV threshold of ≥ 2.5 g/ml, ≥ 4.0 g/ml, > 41% SUVmax, and ≥ 1.5 × liver SUVmean + 2 standard deviations, respectively.

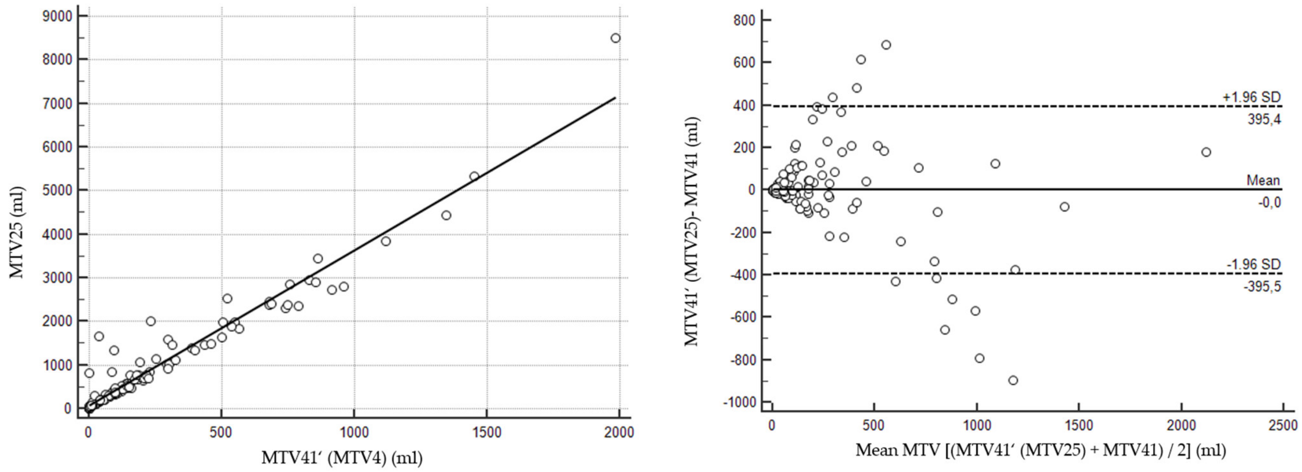

A

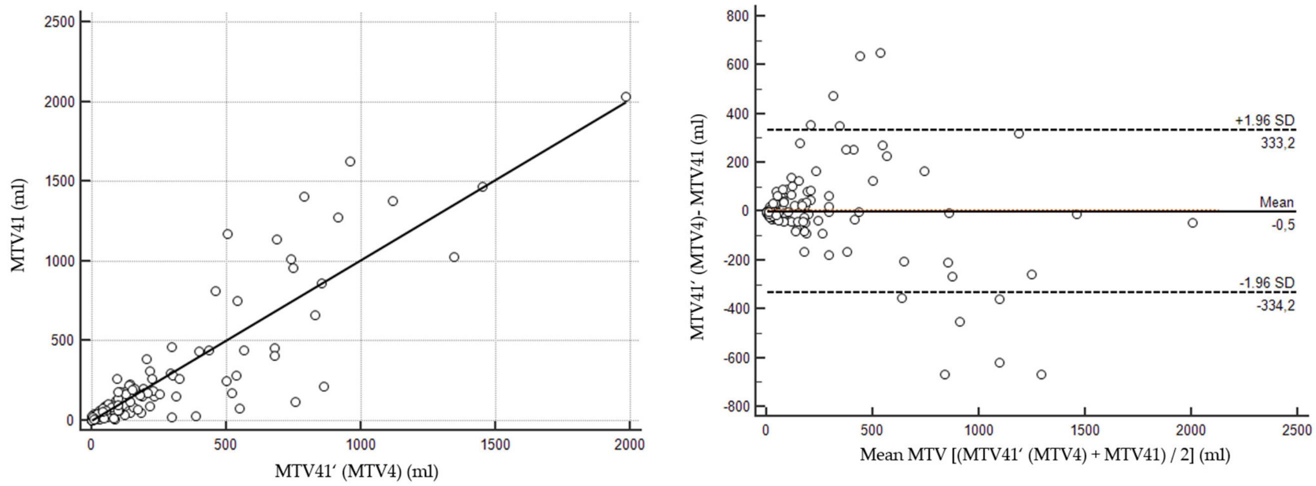

B

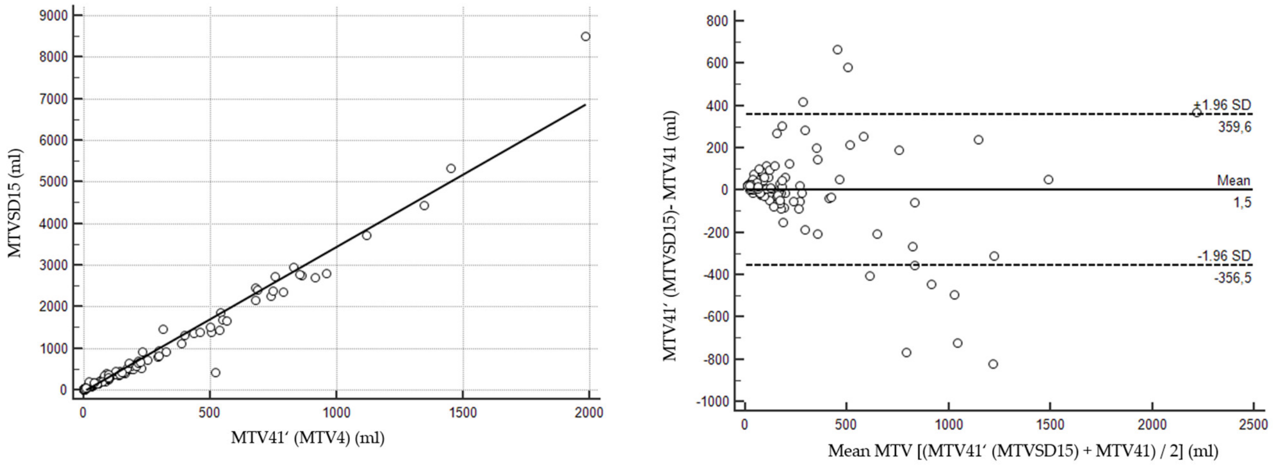

C

Figure S1.2. Regression analysis scatter plots (left column) and Bland−Altman plot analysis with mean and standard deviation (SD) lines (right column) of metabolic tumor volumes (MTV) across three PET threshold methods: MTV25 (row A), MTV41

(row B), and MTVSD15 (row C), calculated using a SUV threshold of ≥ 2.5 g/ml, ≥ 4.0 g/ml, > 41% SUVmax , and ≥ 1.5 × liver SUVmean + 2 standard deviations, respectively, versus regression derived MTV41' based on fixed SUV thresholding method of ≥ 4.0 g/ml.
