# Supplementary material for: Prognostic Value of Multiple Manual Segmentation Methods for Diffuse Large B-Cell Lymphoma with 18F-FDG PET/CT
Source: Curr Oncol. 2025 Jun 16;32(6):356. doi: 10.3390/curroncol32060356 (PMC12191845; doi:10.3390/curroncol32060356)
Supplement: Supplementary file 1 [file curroncol-32-00356-s001.zip › Supplement S2.pdf]

Supplement S2: Receiver-operating-characteristic curves and Kaplan–Meier curves of Progression-free survival in overall cohort (n=140).

Table S2.1. Receiver-operating-characteristic analysis of metabolic tumor volumes (MTVs) determined using various thresholding methods for 2-year progression-free survival (PFS2) prognostication in overall cohort (n=140). MTV25, MTV4, MTV41, MTVSD15: MTVs calculated using a SUV threshold of ≥ 2.5 g/ml, ≥ 4.0 g/ml, > 41% SUVmax, and ≥ 1.5 × liver SUVmean + 2 standard deviations, respectively; LLR, lesion-to-liver ratio; IPI, International Prognostic Index score. * indicates statistical significance at $p < 0.05$. Sensitivity and specificity are reported at optimal cutoffs determined by Youden's index.

	**AUC**	**CI_low**	**CI_high**	**p-Value**	**Sensitivity**	**Specificity**	Optimal threshold value for predicting PFS2
IPI	0.73	0.65	0.80	<0.0001*	64	72	>3
SUVmax	0.50	0.42	0.59	0.9547	48	61	≤24.14
MTV25	0.67	0.59	0.75	0.0045*	84	48	>208 ml
MTV41	0.67	0.59	0.75	0.0035*	56	75	>169 ml
MTV4	0.67	0.58	0.74	0.0074*	80	52	>171 ml
MTVSD15	0.65	0.57	0.73	0.0159*	80	51	>176 ml
TLG25	0.67	0.59	0.75	0.0069*	80	56	>2563
TLG41	0.65	0.57	0.73	0.0151*	80	48	>688
TLG4	0.65	0.57	0.73	0.017*	72	57	>2259
TLGSD15	0.65	0.56	0.73	0.0208*	76	56	>2278
LLR	0.56	0.47	0.64	0.4575	32	90	>20.7

Figure S2.1. Receiver-operating-characteristic curves of metabolic tumor volumes (MTVs) determined using various thresholding methods for 2-year progression-free survival prognostication (PFS2) in overall cohort (n=140). MTV25, MTV4, MTV41, MTVSD15: MTVs calculated using a SUV threshold of ≥ 2.5 g/ml, ≥ 4.0 g/ml, > 41% SUVmax, and ≥ 1.5 × liver SUVmean + 2 standard deviations, respectively; LLR, lesion-to-liver ratio; IPI, International Prognostic Index score. Sensitivity and specificity are reported at optimal cutoffs determined by Youden's index.

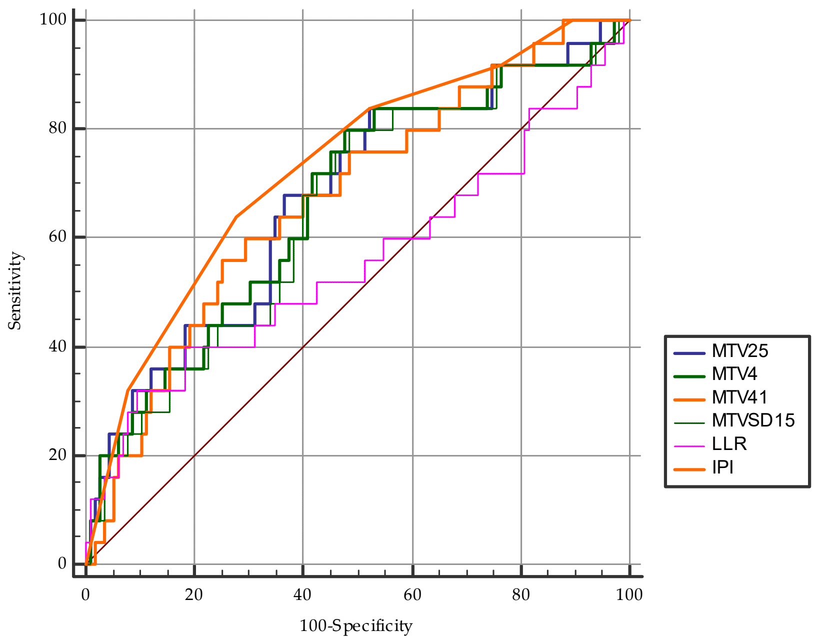

Table S2.2. Kaplan–Meier analysis of metabolic tumor volumes (MTVs) determined using various thresholding methods for 2-year progression-free survival (PFS2) prognostication in overall cohort (n=140). MTV25, MTV4, MTV41, MTVSD15: MTVs calculated using a SUV threshold of ≥ 2.5 g/ml, ≥ 4.0 g/ml, > 41% SUVmax, and ≥ 1.5 × liver SUVmean + 2 standard deviations, respectively; LLR, lesion-to-liver ratio; IPI, International Prognostic Index score. * indicates statistical significance at $p < 0.05$.

	**p-Value**	**HR**	**95%CI**
MTV25	0.0013*	3,46	1.62 – 7.38
MTV4	0.0012*	3,5	1.64 – 7.44
MTV41	0.0005*	4,36	1.9 – 10.01
MTVSD15	0.0015*	3,39	1.59 – 7.21
IPI	<0.0001*	5,57	2.45 – 12.66
LLR	0.0042*	5,52	1.71 – 17.76

Figure S2.2. Kaplan–Meier curves of 2-year progression-free survival in overall cohort (n=140) by MTV25, MTV4, MTV41, MTVSD15, LLR, and IPI: MTVs calculated using a SUV threshold of ≥ 2.5 g/ml, ≥ 4.0 g/ml, > 41% SUVmax, and ≥ 1.5 × liver SUVmean + 2 standard deviations, respectively; LLR, lesion-to-liver ratio; IPI, International Prognostic Index score.

Kaplan-Meier Estimates of Two-Year Progression-Free Survival by MTV25 Groups

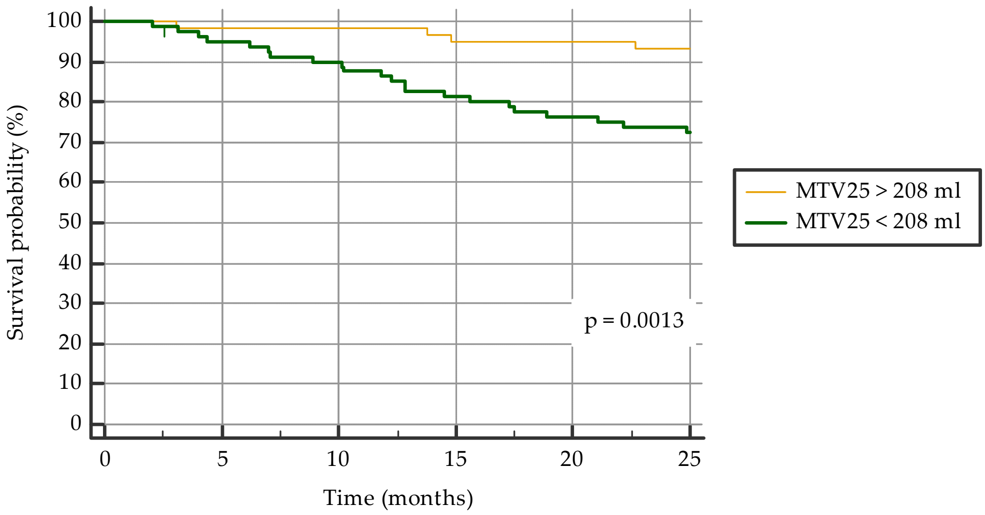

Number at risk

Group	0	5	10	15	20	25
Group: MTV25 > 208 ml	59	58	58	56	56	55
Group: MTV25 < 208 ml	81	76	72	65	61	58

Kaplan-Meier Estimates of Two-Year Progression-Free Survival by MTV4 Groups

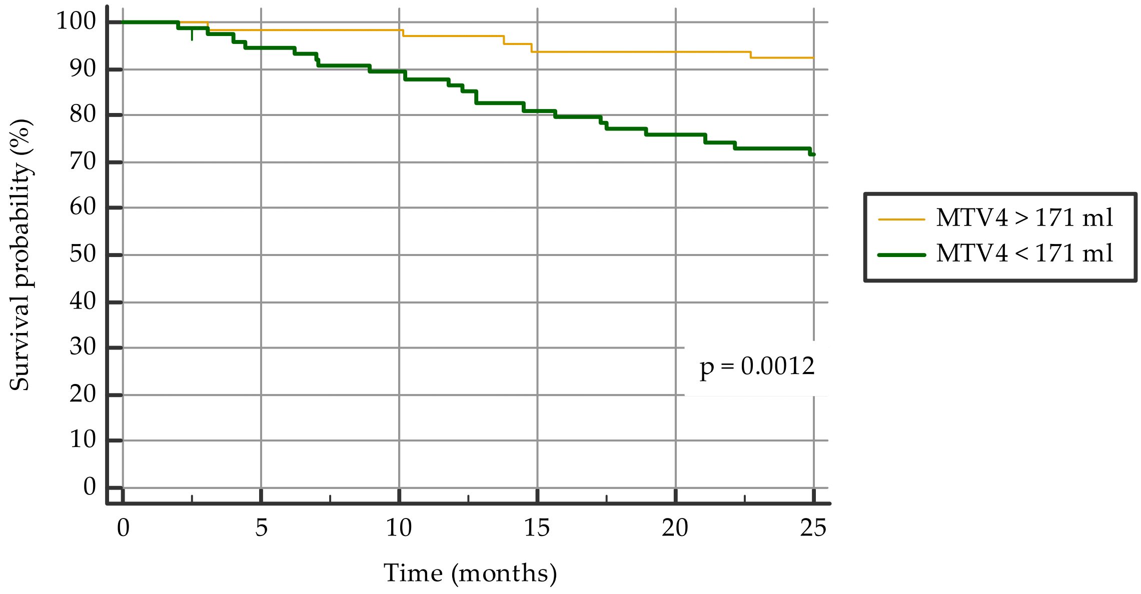

Number at risk

Group	0	5	10	15	20	25
Group: MTV4 > 171 ml	65	64	64	61	61	60
Group: MTV4 < 171 ml	75	70	66	60	56	53

Kaplan-Meier Estimates of Two-Year Progression-Free Survival by MTV41 Groups

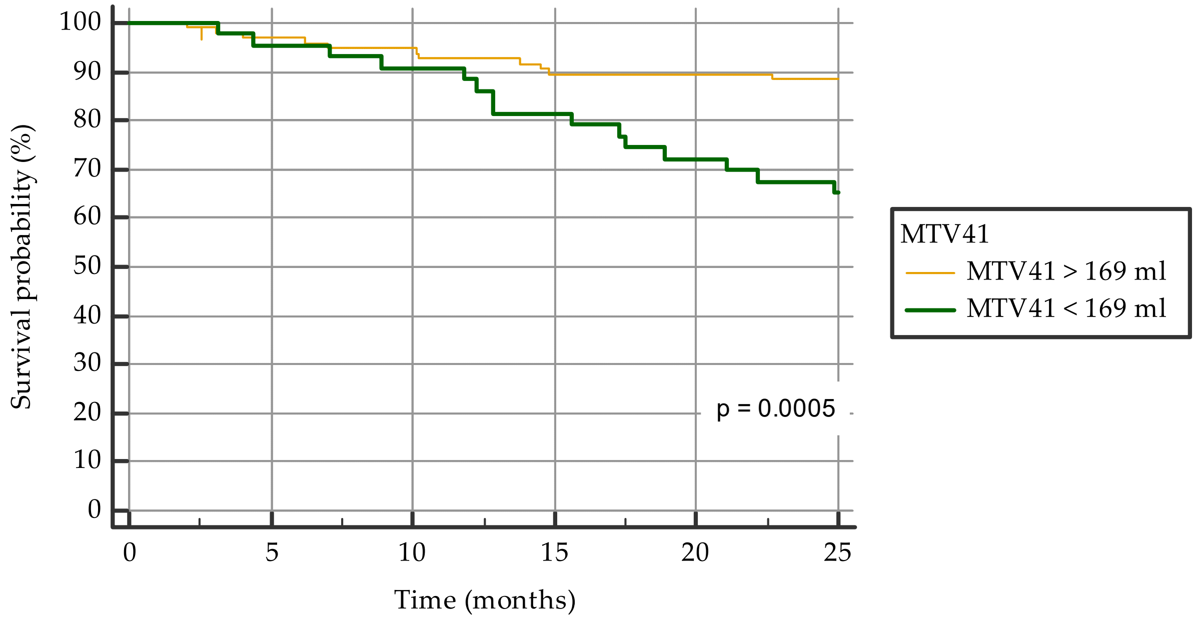

Number at risk

	0	5	10	15	20	25
Group: MTV41 > 169 ml	97	93	91	86	86	85
Group: MTV41 < 169 ml	43	41	39	35	31	28

Kaplan-Meier Estimates of Two-Year Progression-Free Survival by MTVSD15 Groups

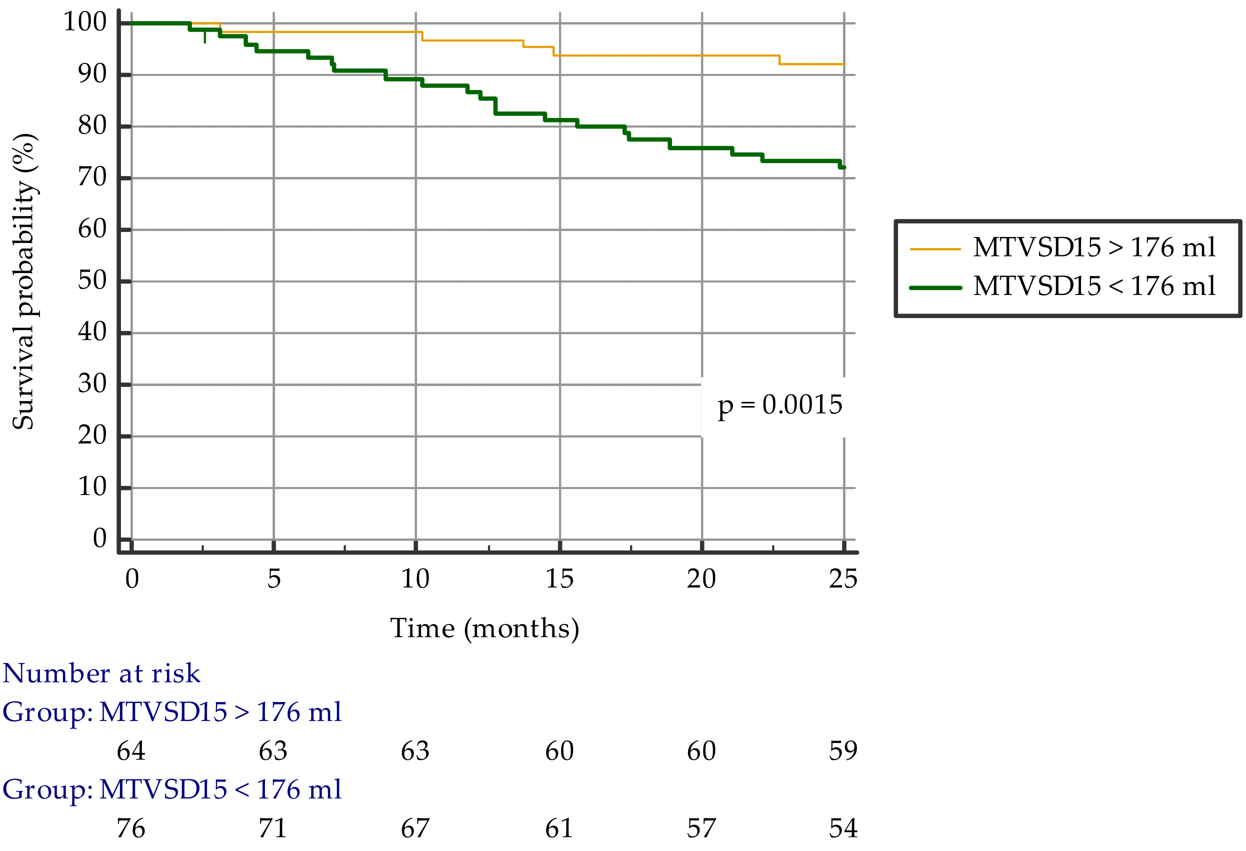

Kaplan-Meier Estimates of Two-Year Progression-Free Survival by LLR Groups

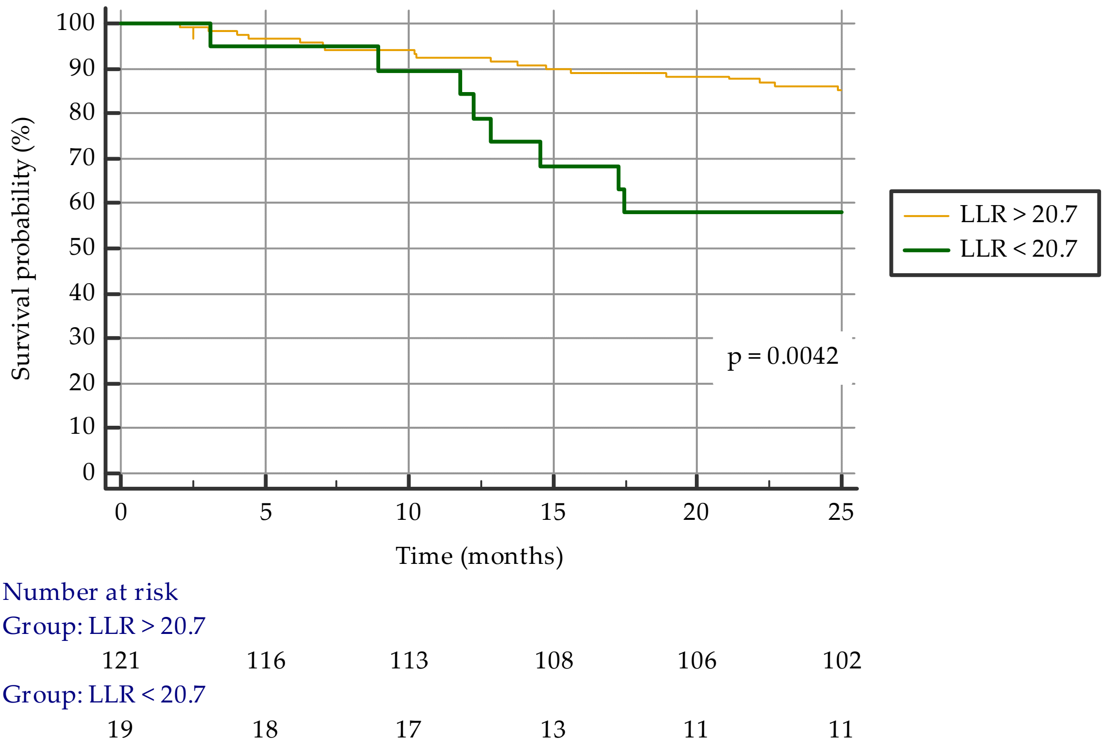

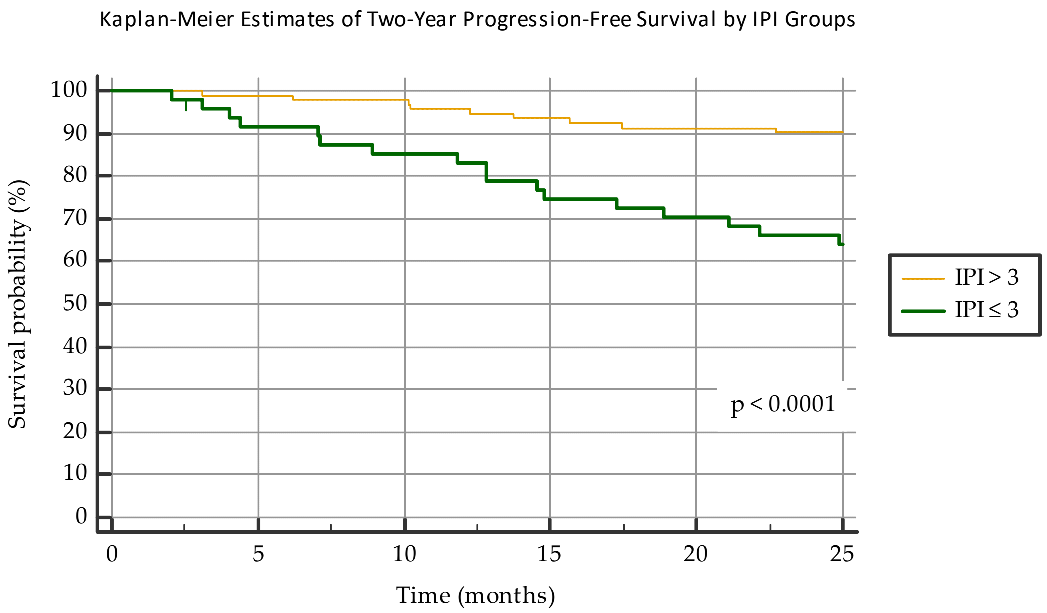

Number at risk

Group: IPI > 3						
	92	91	90	86	84	83
Group: IPI ≤ 3						
	48	43	40	35	33	30
