# Supplementary material for: Prognostic Value of Multiple Manual Segmentation Methods for Diffuse Large B-Cell Lymphoma with 18F-FDG PET/CT
Source: Curr Oncol. 2025 Jun 16;32(6):356. doi: 10.3390/curroncol32060356 (PMC12191845; doi:10.3390/curroncol32060356)
Supplement: Supplementary file 1 [file curroncol-32-00356-s001.zip › Supplement S3.pdf]

Supplement S3. Receiver-operating-characteristic curves and Kaplan–Meier curves of overall survival in overall cohort (n=140).

Figure S3.1. Receiver-operating-characteristic curves of metabolic tumor volumes (MTVs) determined using various thresholding methods for 3-year overall survival prognostication in all patients (n=140). MTV25, MTV4, MTV41, MTVSD15: MTVs calculated using a SUV threshold of ≥ 2.5 g/ml, ≥ 4.0 g/ml, > 41% SUVmax, and ≥ 1.5 × liver SUVmean + 2 standard deviations, respectively; LLR, lesion-to-liver ratio; IPI, International Prognostic Index score.

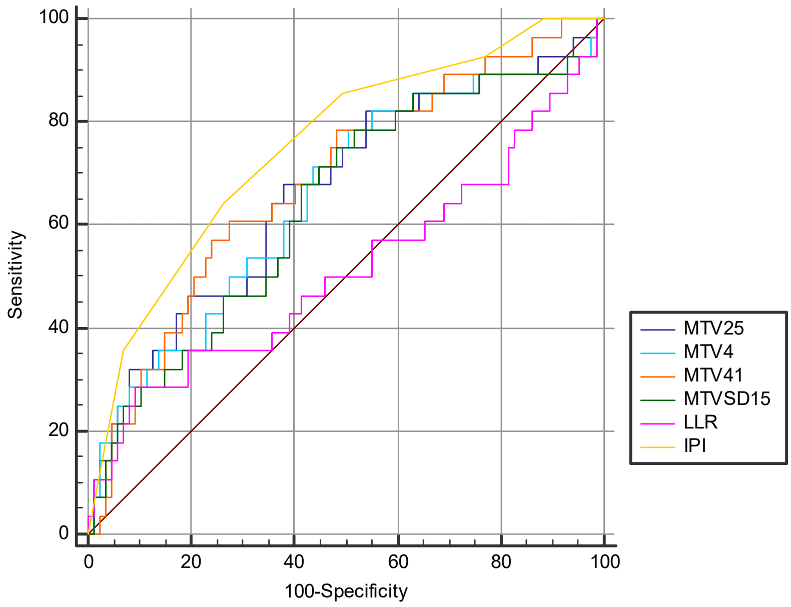

Figure S3.2. Kaplan–Meier curves of overall survival in all patients (n=140) by MTV25, MTV4, MTV41, MTVSD15, LLR, SUVmax, and IPI: MTVs calculated using a SUV threshold of ≥ 2.5 g/ml, ≥ 4.0 g/ml, > 41% SUVmax, and ≥ 1.5 × liver SUVmean + 2 standard deviations, respectively; LLR, lesion-to-liver ratio; IPI, International Prognostic Index score.

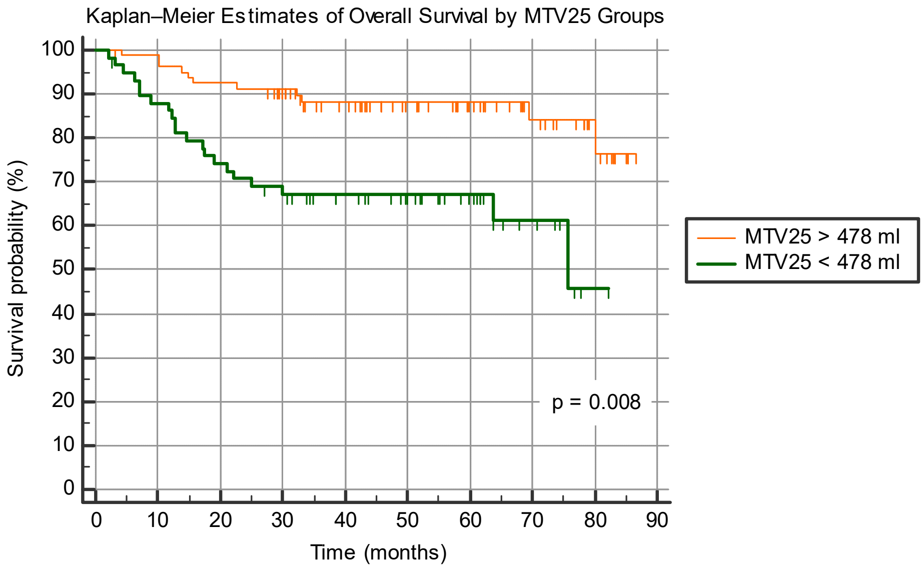

Number at risk

Group: MTV25 > 478 ml										
	81	79	74	65	53	40	30	20	11	0
Group: MTV25 < 478 ml										
	59	51	43	38	31	24	16	7	1	0

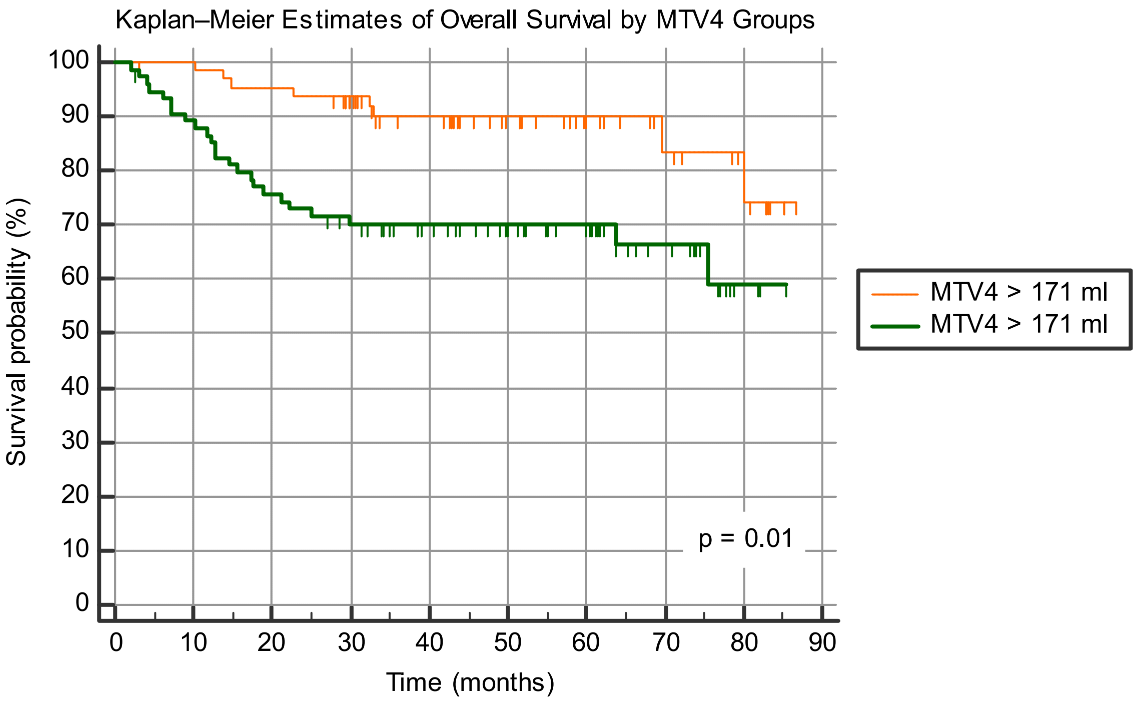

Number at risk

Group: MTV4 > 171 ml

65	64	61	53	43	32	21	13	9	0

Group: MTV4 > 171 ml

75	66	56	50	41	32	25	14	3	0

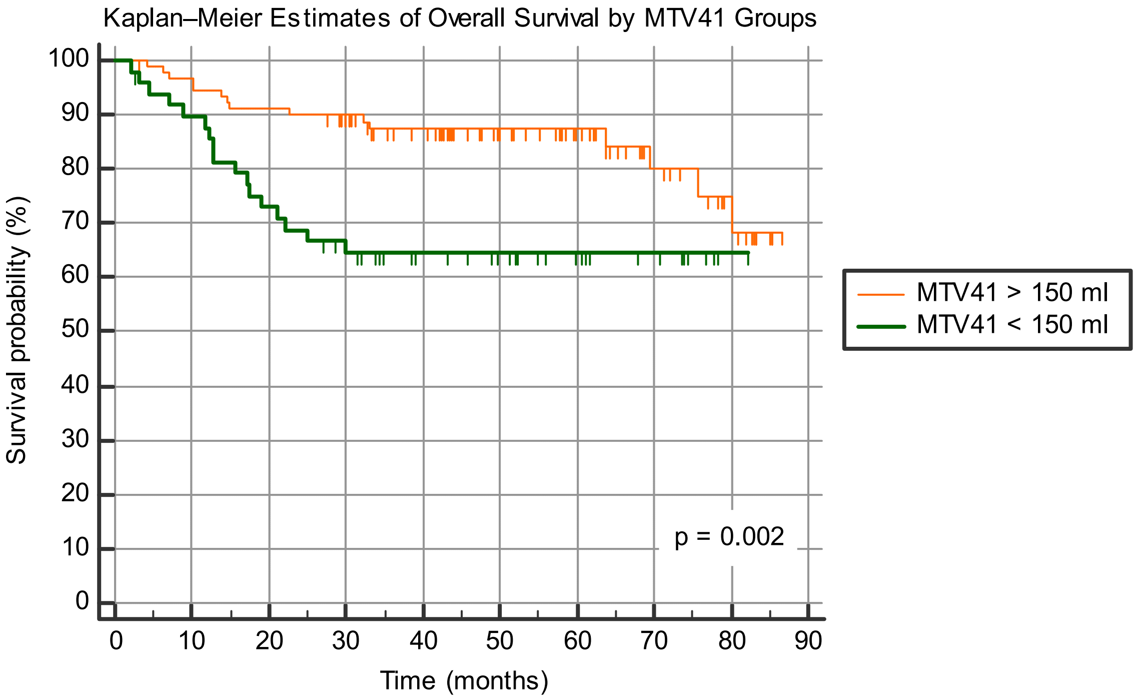

Number at risk

Group: MTV41 > 150 ml

91	87	82	74	62	46	34	19	11	0

Group: MTV41 < 150 ml

49	43	35	29	22	18	12	8	1	0

Kaplan–Meier Estimates of Overall Survival by MTVSD15 Groups

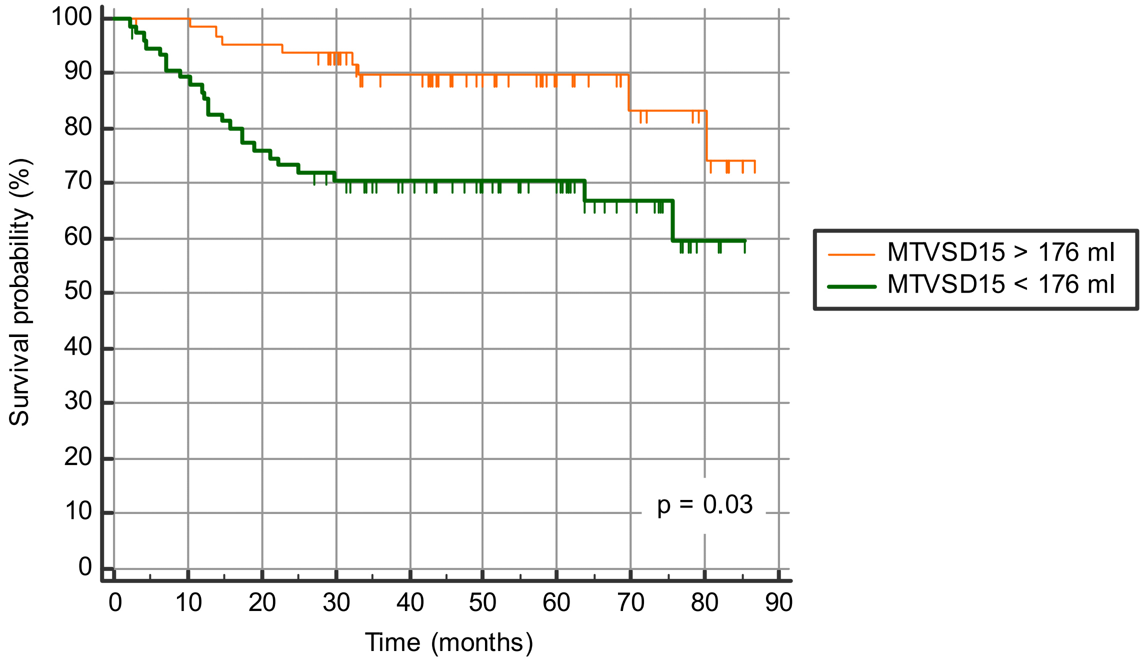

Number at risk

Group: MTVSD15 > 176 ml

64	63	60	52	42	31	20	13	9	0

Group: MTVSD15 < 176 ml

76	67	57	51	42	33	26	14	3	0

Kaplan–Meier Estimates of Overall Survival by LLR Groups

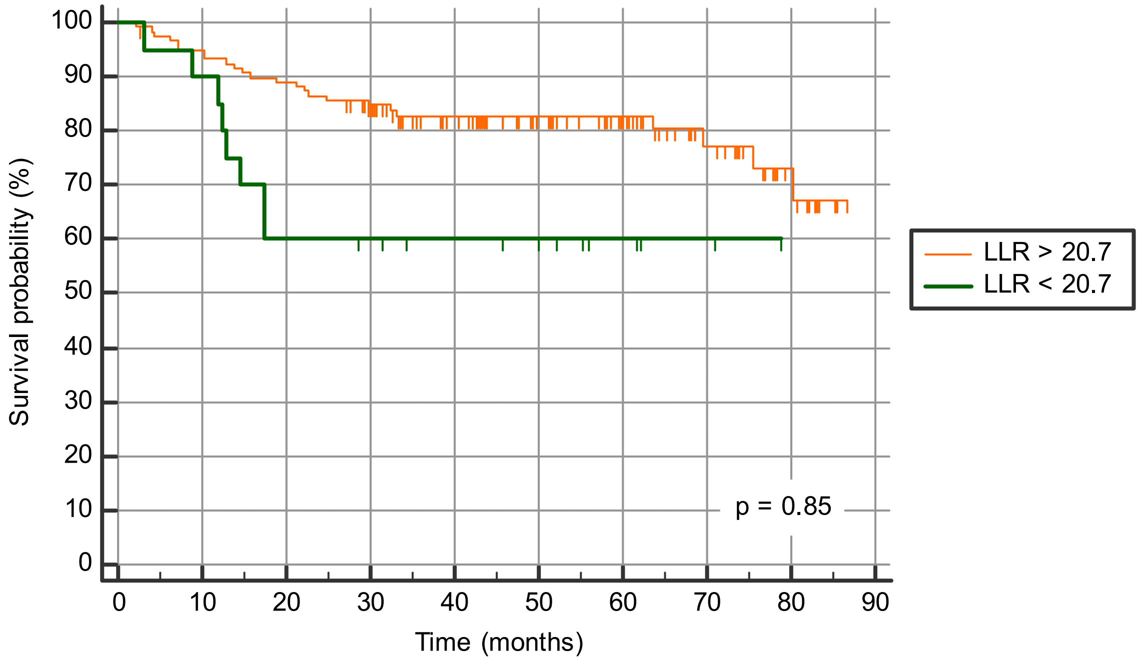

Number at risk

Group: LLR > 20.7

120	112	105	92	75	57	42	25	12	0

Group: LLR < 20.7

20	18	12	11	9	7	4	2	0	0

Kaplan-Meier Estimates of Overall Survival by SUVmax Groups

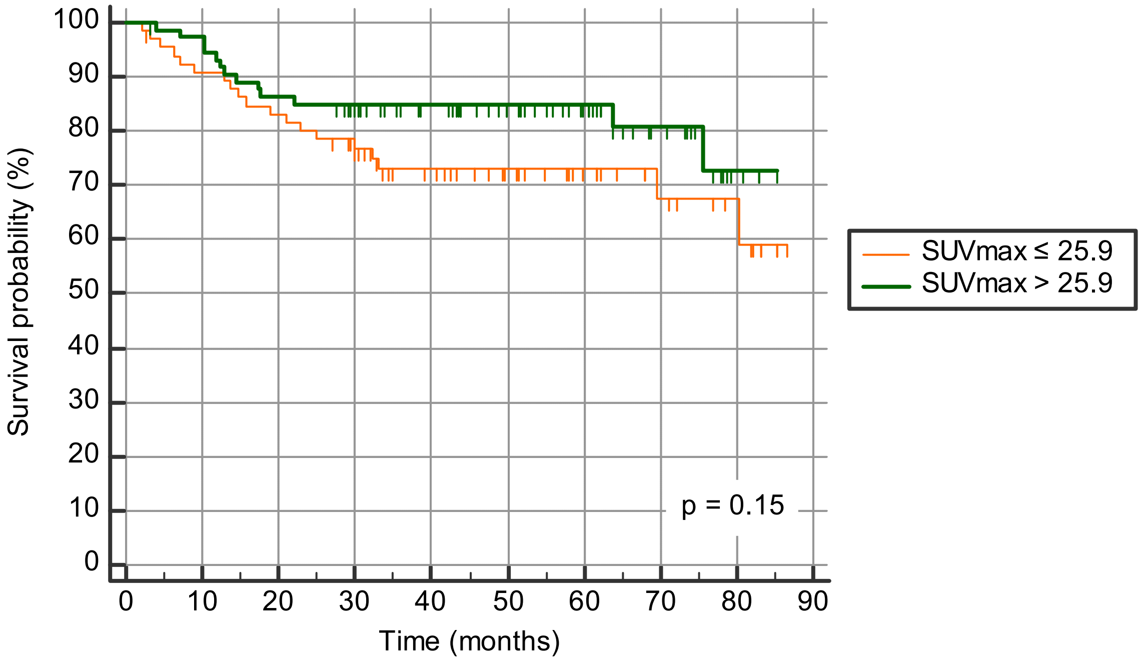

Number at risk

Group: SUVmax ≤ 25.9									
66	59	54	46	36	27	19	12	8	0
Group: SUVmax > 25.9									
74	71	63	57	48	37	27	15	4	0

Kaplan–Meier Estimates of Overall Survival by IPI Groups

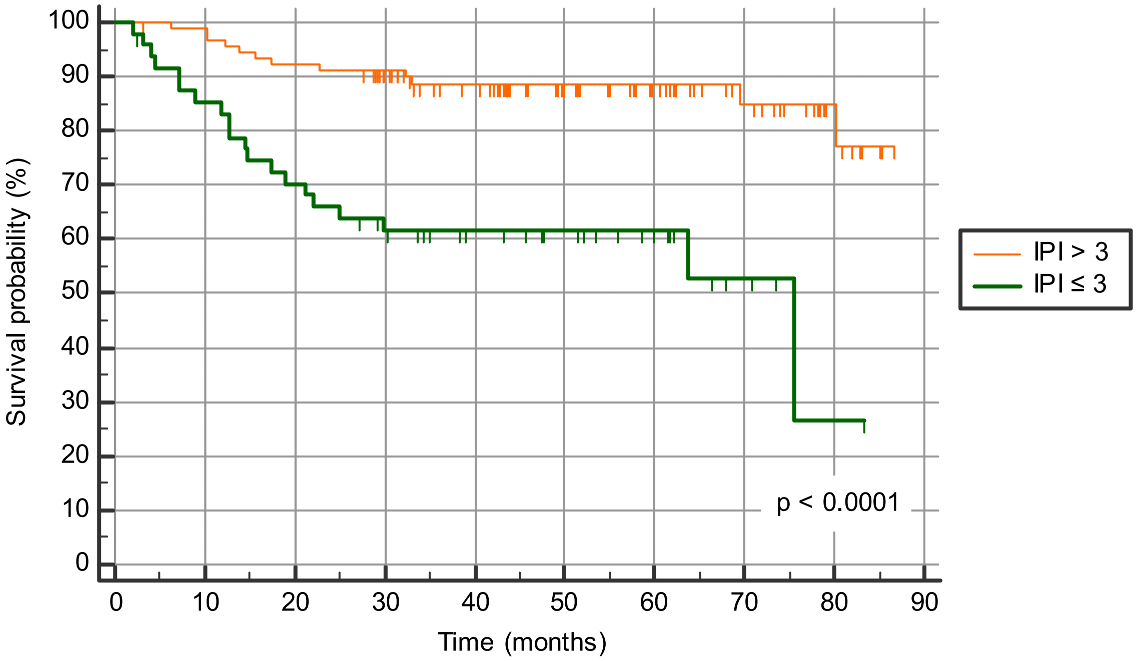

Number at risk

Group: IPI > 3									
92	90	84	76	63	47	36	23	11	0
Group: IPI ≤ 3									
48	40	33	27	21	17	10	4	1	0
