# Supplementary material for: Prognostic Value of Multiple Manual Segmentation Methods for Diffuse Large B-Cell Lymphoma with 18F-FDG PET/CT
Source: Curr Oncol. 2025 Jun 16;32(6):356. doi: 10.3390/curroncol32060356 (PMC12191845; doi:10.3390/curroncol32060356)
Supplement: Supplementary file 1 [file curroncol-32-00356-s001.zip › Supplement S4.pdf]

Supplement S4. Median values of variables, Receiver-operating-characteristic curves and Kaplan–Meier curves of overall survival in stage 4 patient subgroup (n=84).

Table S4. The median values of variables in the stage 4 patient subgroup

	median	95%CI of median	range	IQR
SUVmax	27.90	24.1 - 29.7	6.3 - 55.4	20.3 - 33.6
MTV25 [ml]	652.4	383.7 - 875.6	2.9 - 8498.1	205.9 - 1862.4
MTV4 [ml]	410.6	240.1 - 579.6	0.6 - 5243.3	110.8 - 1292.9
MTV41 [ml]	85.5	61.0 - 159.5	0.7 - 2031.0	25.3 - 339.5
MTVSD15 [ml]	398.6	274.8 - 609.1	0.9 - 8498.1	143.3 - 1381.8
LLR	13.5	12.0 - 15.2	2.9 - 35.5	11.0 - 19.1
TLG25	4724.2	2990.5 - 7856.8	10.5 - 43106.2	1333.3 - 12364.8
TLG4	3851.8	2182.3 - 6406.5	3.1 - 37128.9	1013.5 - 11667.4
TLG41	1545.8	995.2 - 2295.4	10.0 - 27069.3	361.7 - 6387.6
TLGSD15	3926.6	2417.9 - 5900.9	4.3 - 43106.2	993.8 - 12017.9
IPI	4	3 - 4	2 - 5	3 - 4

95%CI, 95% confidence intervals; IQR, Interquartile range; MTV25, MTV4, MTV41, MTVSD15, TLG25, TLG4, TLG41, TLGSD15: MTVs and TLGs calculated using a SUV threshold of ≥ 2.5 g/ml, ≥ 4.0 g/ml, > 41%SUVmax, and ≥ 1.5 × liver SUVmean + 2 standard deviations, respectively. LLR, lesion-to-liver ratio; IPI, International Prognostic Index score.

Figure S4.1. Receiver-operating-characteristic curves of metabolic tumor volumes (MTVs) determined using various thresholding methods for 3-year overall survival prognostication in the stage 4 patient subgroup (n=84). MTV25, MTV4, MTV41, MTVSD15: MTVs calculated using a SUV threshold of ≥ 2.5 g/ml, ≥ 4.0 g/ml, > 41% SUVmax, and ≥ 1.5 × liver SUVmean + 2 standard deviations, respectively; LLR, lesion-to-liver ratio; IPI, International Prognostic Index score.

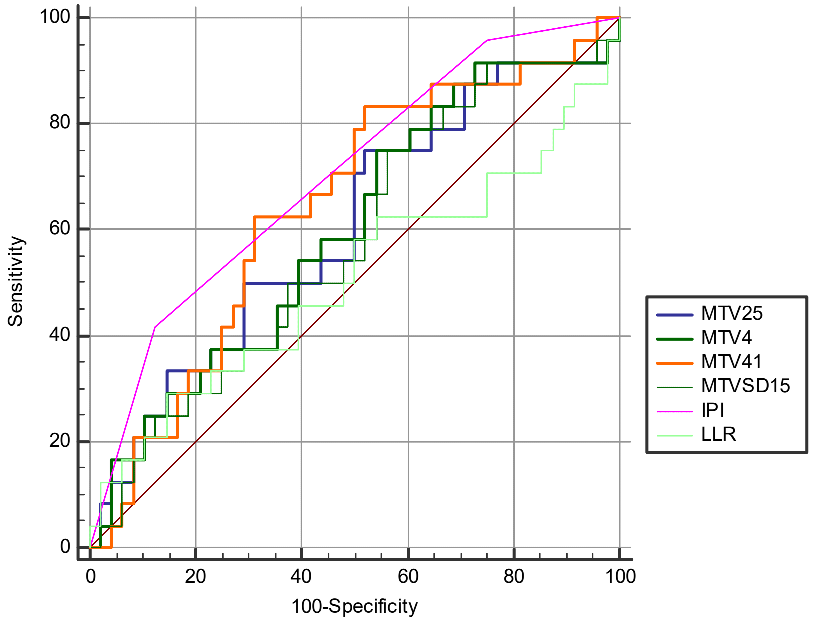

Figure S4.2. Kaplan–Meier curves of overall survival in the stage 4 patient subgroup by MTV25, MTV4, MTV41, MTVSD15, LLR, SUVmax, and IPI: MTVs calculated using a SUV threshold of ≥ 2.5 g/ml, ≥ 4.0 g/ml, > 41% SUVmax, and ≥ 1.5 × liver SUVmean + 2 standard deviations, respectively; LLR, lesion-to-liver ratio; IPI, International Prognostic Index score.

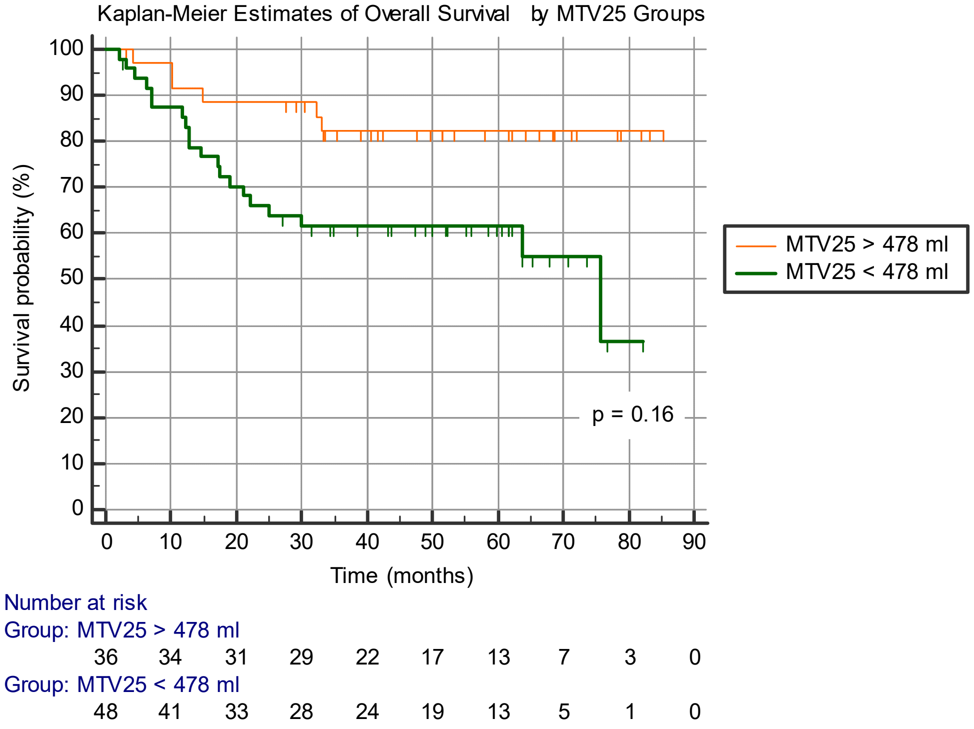

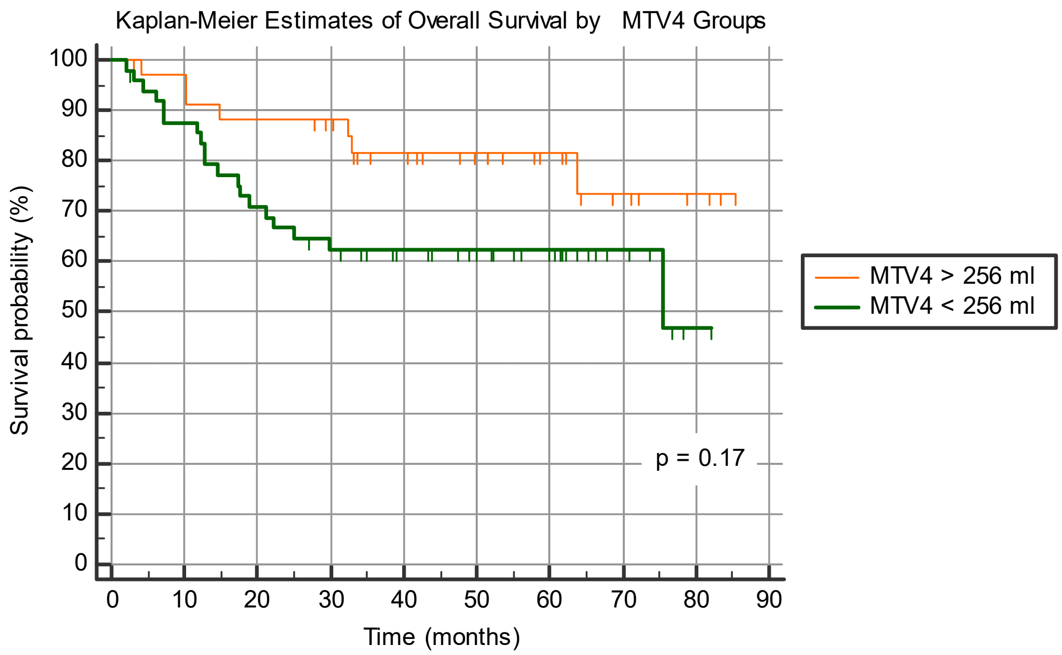

Number at risk

Group: MTV4 > 256 ml

35	33	30	28	22	17	12	6	3	0

Group: MTV4 < 256 ml

49	42	34	29	24	19	14	6	1	0

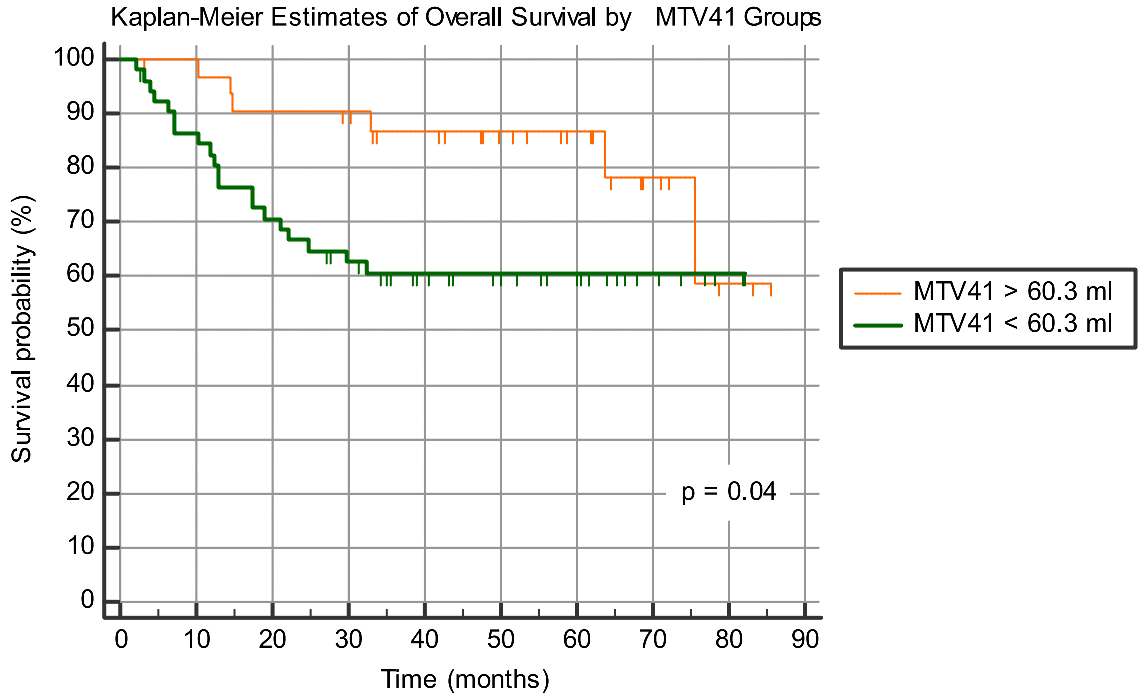

Number at risk

Group: MTV41 > 60.3 ml

32	31	28	27	23	18	13	6	2	0

Group: MTV41 < 60.3 ml

52	44	36	30	23	18	13	6	2	0

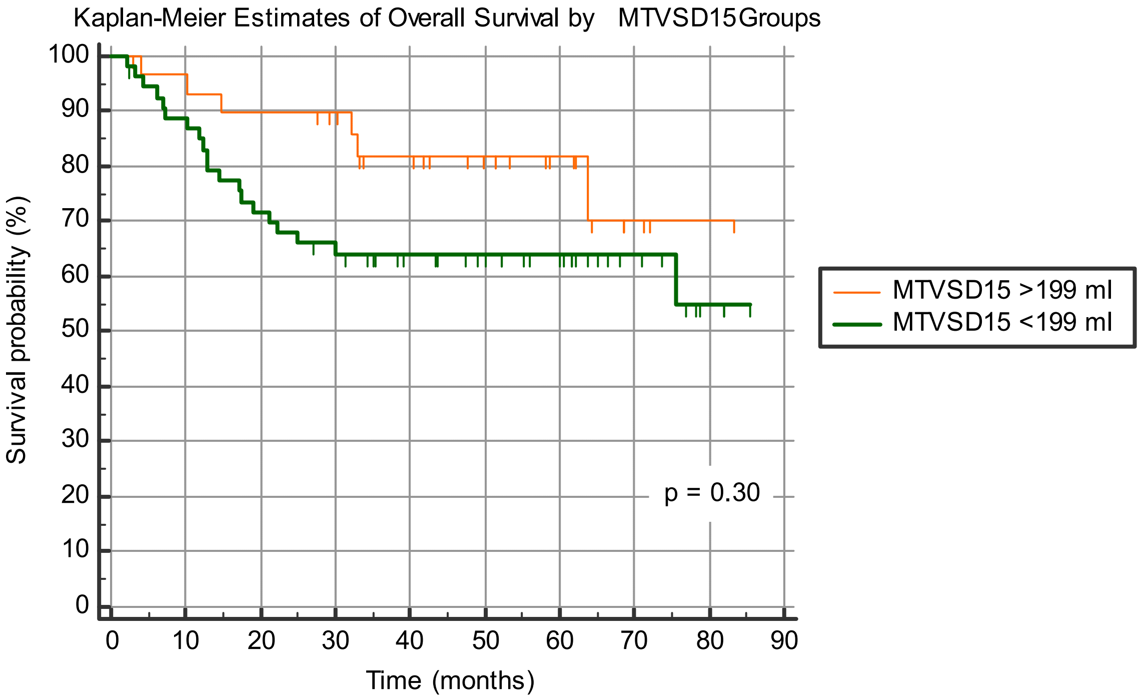

Number at risk

Group: MTVSD15 >199 ml

30	28	26	24	19	14	9	3	1	0

Group: MTVSD15 <199 ml

54	47	38	33	27	22	17	9	3	0

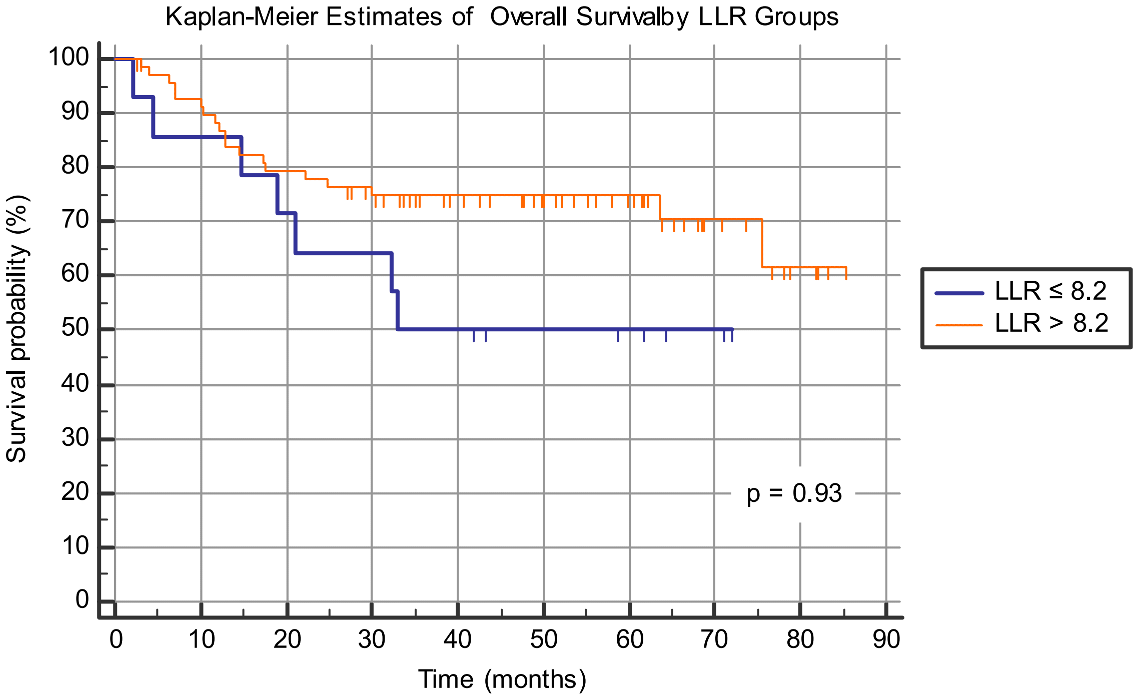

Number at risk

Group: LLR ≤ 8.2

14	12	10	9	7	5	4	2	0	0

Group: LLR > 8.2

70	63	54	48	39	31	22	10	4	0

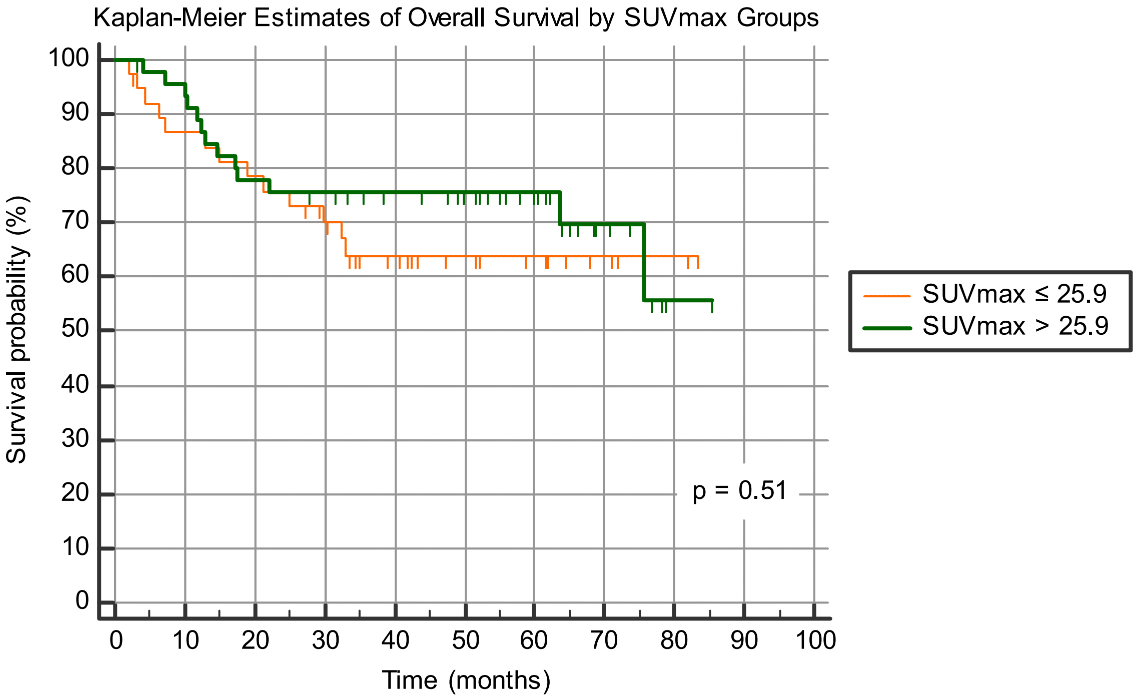

Number at risk

Group: SUVmax ≤ 25.9

38	32	29	24	17	12	9	5	3	0

Group: SUVmax > 25.9

46	43	35	33	29	24	17	7	1	0

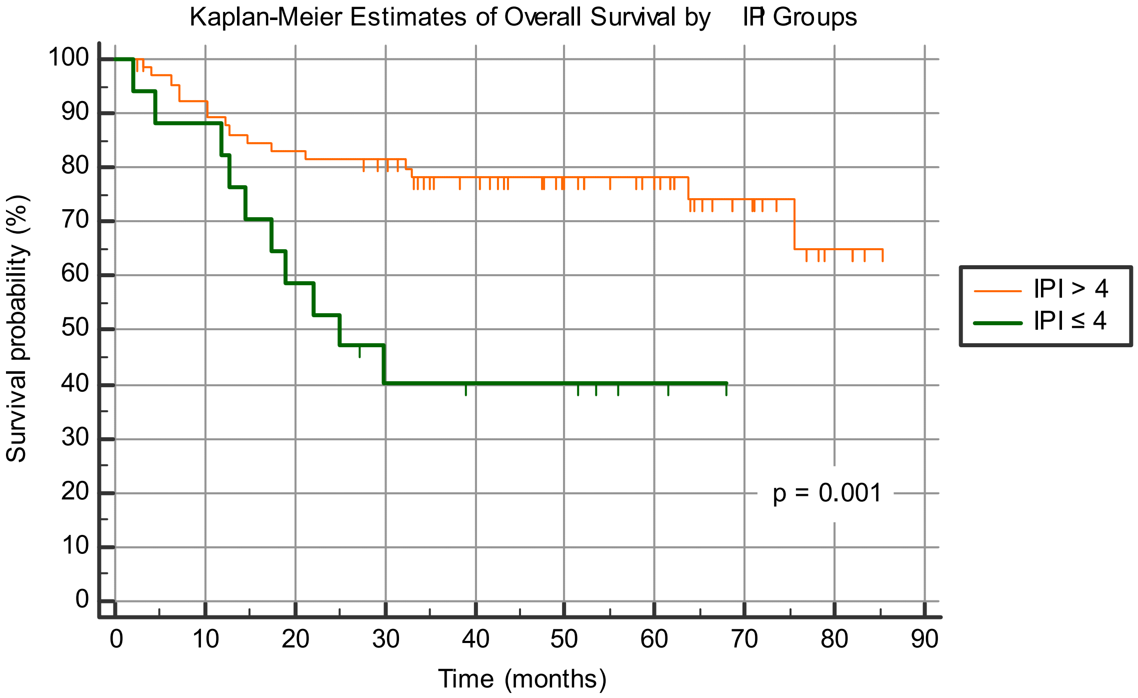

Number at risk

Group: IPI > 4

67	60	54	51	41	31	24	12	4	0

Group: IPI ≤ 4

17	15	10	6	5	5	2	0	0	0
